# Neutrophil Function in Patients With Chronic Kidney Disease: A Systematic Review and Meta‐Analysis

**DOI:** 10.1111/apha.70057

**Published:** 2025-05-24

**Authors:** Jane Sophie Lauxen, Sonja Vondenhoff, Carolina Victoria Cruz Junho, Philipp Martin, Susanne Fleig, Katharina Schütt, Ulrike Schulze‐Späte, Oliver Soehnlein, Leticia Prates‐Roma, Yvonne Döring, Constance C. F. M. J. Baaten, Heidi Noels

**Affiliations:** ^1^ Institute for Molecular Cardiovascular Research (IMCAR) RWTH Aachen University Aachen Germany; ^2^ Department of Nephrology and Clinical Immunology RWTH Aachen University Aachen Germany; ^3^ Department of Internal Medicine I—Cardiology, Angiology and Internal Intensive Care Medicine RWTH Aachen University Hospital Aachen Germany; ^4^ Section of Geriodontics, Department of Conservative Dentistry and Periodontics University Hospital Jena Jena Germany; ^5^ Institute of Experimental Pathology (ExPat) Center for Molecular Biology of Inflammation (ZMBE), University Hospital Münster, University of Münster Münster Germany; ^6^ Biophysics, Center for Integrative Physiology and Molecular Medicine (CIPMM), Center for Human and Molecular Medicine (ZHMB), Center for Gender‐Specific Biology and Medicine (CGBM), Faculty of Medicine Saarland University Homburg Germany; ^7^ Division of Angiology, Swiss Cardiovascular Center, Inselspital, Bern University Hospital University of Bern Bern Switzerland; ^8^ Department for BioMedical Research (DBMR) University of Bern Bern Switzerland; ^9^ DZHK (German Centre for Cardiovascular Research), Partner Site Munich Heart Alliance Munich Germany; ^10^ Institute for Cardiovascular Prevention (IPEK), Ludwig‐Maximilians‐University Munich (LMU) Munich Germany; ^11^ Department of Biochemistry, CARIM, Cardiovascular Research Institute Maastricht Maastricht University Maastricht the Netherlands; ^12^ Aachen‐Maastricht Institute for Cardiorenal Disease (AMICARE) University Hospital RWTH Aachen Aachen Germany

**Keywords:** chronic kidney disease, neutrophils, reactive oxygen species

## Abstract

**Background:**

Patients with chronic kidney disease (CKD) are at increased cardiovascular risk. Since neutrophils play a central role in atherosclerosis and cardiovascular disease, this study analyzed neutrophil function in CKD patients.

**Methods:**

A systematic review of neutrophil function in CKD patients compared to controls was performed according to PRISMA guidelines by searching PubMed and the Web of Science. A meta‐analysis summarized the production of reactive oxygen species (ROS) in CKD patients on dialysis in Forest plots. Influencer outlier analyses evaluated risk of bias.

**Results:**

Overall, 92 studies were included, of which 18 in the meta‐analysis. Although study heterogeneity was high, the systematic review identified primarily reduced phagocytosis capacity but increased neutrophil degranulation and basal ROS production in neutrophils from CKD patients on hemodialysis compared to controls. Phagocytosis and basal ROS production were mainly unaltered in non‐dialysis dependent CKD patients and CKD patients on peritoneal dialysis. The meta‐analysis confirmed increased ROS generation in basal conditions predominantly in CKD patients on hemodialysis (Hedges *g* = 1.20, 95% CI: [0.32; 2.09]), with an insufficient study number for a clear comparison to CKD patients on peritoneal dialysis. However, upon neutrophil stimulation with sterile inflammatory triggers, ROS production was also increased in neutrophils from patients on peritoneal dialysis (Hedges *g* = 0.89, 95% CI: [0.34; 1.43]).

**Conclusion:**

Increased degranulation and basal ROS formation were observed in neutrophils of CKD patients on hemodialysis, which could contribute to their increased cardiovascular risk. Future studies should compare neutrophil activity in patients of different CKD stages and comorbidities also in relation to cardiovascular outcomes.

**Abbreviations**
CI: confidence interval
CKD: chronic kidney disease
CVD: cardiovascular disease
fMLP: N‐formylmethionyl‐leucyl‐phenylalanine (officially called fMLF)
HD: hemodialysis
MPO: myeloperoxidase
NETs: neutrophil extracellular traps
NOX: NADPH‐oxidase
PD: peritoneal dialysis
PMA: Phorbol 12‐myristate 13‐acetate
ROS: reactive oxygen species
TNF: tumor necrosis factor

## 1 Introduction

Chronic kidney disease (CKD) is characterized by kidney damage or a reduced kidney filtration function for more than 3 months and has a substantial impact on health and survival outcomes [1]. It has a global prevalence of ~13.4% [2] and a high socio‐economic and environmental burden. CKD is a progressive disease and is classified in CKD stages 1–5 based on the remaining glomerular filtration rate (GFR): patients can present with a normal to mild kidney dysfunction (CKD1‐2), a mild–moderate or moderate–severe kidney dysfunction (CKD3a/b), a severe kidney impairment (CKD4) or complete kidney failure (CKD5) [3]. In this last stage, patients require kidney replacement therapy such as dialysis (hemodialysis (HD) or peritoneal dialysis (PD)) and kidney transplantation for survival. However, even with these interventions, life expectancy remains shortened, particularly due to an increase in cardiovascular risk that starts already in the early stages of CKD and grows exponentially with the progression of kidney dysfunction [4].

Overall, CKD patients show a chronic low‐grade inflammation and innate immune activation [5], leading to progressive atherosclerosis [6, 7] and an increased risk of cardiovascular and thrombotic complications [4, 6, 8, 9]. Neutrophil granulocytes are the most abundant leukocytes in the blood and play an important role in cardiovascular disease (CVD) including atherosclerosis and thrombotic risk through the production of reactive oxygen species (ROS), the release of pro‐inflammatory molecules (e.g., neutrophil elastase and myeloperoxidase) and the formation of neutrophil extracellular traps (NETs) [10, 11]. Increased neutrophil numbers [12, 13] and alterations in neutrophil activation responses have been reported [10] in CKD, though with a wide variety in readouts. Therefore, we executed a systematic review on the effects of CKD on neutrophil phenotype and function. We also performed a meta‐analysis of neutrophil ROS production in CKD patients, since this was the most frequently studied readout of neutrophil responses in CKD and because of the critical role of ROS production in the context of CVD.

## 2 Methods

This study was performed according to the Preferred Reporting Items for Systematic reviews and Meta‐Analyses (PRISMA) guidelines.

### 2.1 Systematic Review: Search Strategy, Eligibility Criteria and Data Extraction

**FIGURE 1:**
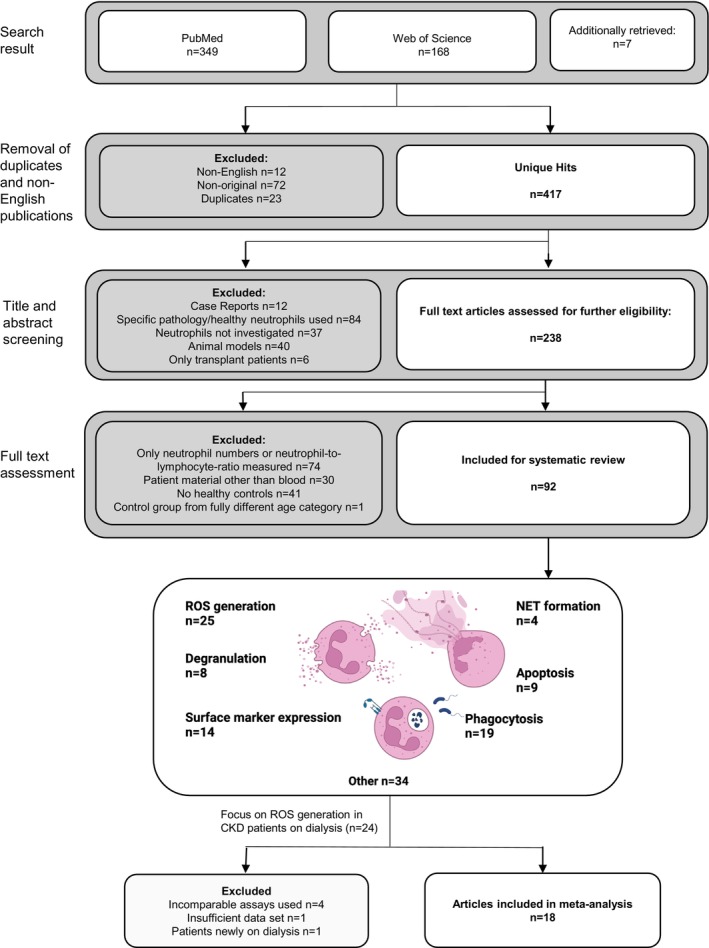
Flow chart of study selection for systematic review and meta‐analysis of neutrophil function in CKD. CKD, chronic kidney disease; NET, neutrophil extracellular traps; ROS, reactive oxygen species.

PubMed and the Web of Science were searched for studies describing the effects of CKD on human neutrophil function published 01/1990–09/2023, with Table S1 providing full details on the systematic search strategy as well as in‐ and exclusion criteria for both the systematic review and the meta‐analysis. In short, studies were considered eligible when the phenotype or function of peripheral blood neutrophils was assessed in CKD patients in comparison to healthy volunteers, with readouts including—but not restricted to—surface activation marker expression, degranulation, ROS production, NET formation, apoptosis, and phagocytosis, and this both in basal as well as stimulated conditions (Figure 1). Studies were selected independently by two reviewers (J.S.L., P.M.) based on the predefined eligibility criteria (Table S1, Figure 1), with eventual disagreements resolved by consulting a third reviewer (H.N.). Additional relevant publications were identified by crosschecking the reference lists of the retrieved studies and relevant reviews (J.S.L., H.N.).

Data extracted from full‐text articles include patient characteristics and study size, as well as findings on neutrophil apoptosis, activation markers, phagocytic activity, ROS production, degranulation, and NET release (Tables S2–S8, J.S.L., double‐checked by C.V.C.J.).

### 2.2 Meta‐Analysis: Data Extraction and Quality Assessment

Since ROS production (i) was the most studied readout, (ii) contributes to CVD pathophysiology, and (iii) showed a high data variability in the systematic review, a meta‐analysis with different subgroup analyses was performed for neutrophil ROS production in CKD. The meta‐analysis could only be performed in CKD patients on dialysis compared to healthy controls, given the insufficient number of datasets for non‐dialysis dependent CKD patients. Studies measured intracellular ROS (by flow cytometric analysis of 2′,7′‐dichlorodihydrofluorescein diacetate (DCFH‐DA) or dihydrorhodamine oxidation), extracellular ROS (superoxide dismutase (SOD)‐inhibitable cytochrome C reduction, lucigenin‐enhanced chemiluminescence) or total ROS (luminol‐enhanced chemiluminescence, dihydroethidium assays and DCFH‐DA assays using plate readers, without addition of membrane‐impermeable ROS scavengers) [14].

For the meta‐analysis, study quality assessment included checking the completeness of the description of the patient population and the methods. Study size and values with mean and standard deviation (SD) or standard error of the mean (SEM) were extracted from the manuscript text, tables or graphical representations (J.S.L., double‐checked by S.V./P.M.). SEM was converted to SD for inclusion in the meta‐analysis. Furthermore, an N‐number correction for multiple comparisons was applied where appropriate (i.e., when multiple CKD cohorts were compared to the same control group or when the same parameter (e.g., intracellular ROS) was measured in different ways).

### 2.3 Meta‐Analysis: Calculation and Statistical Analysis

Statistical analysis and graphical representation were performed in Rstudio v1.3.1093 using the ‘meta’, ‘metafor’ and ‘dmetar’ packages [15, 16]. The effect size of neutrophil ROS production in CKD patients vs. controls was calculated as standardized mean difference using Hedge's g with 95% confidence intervals. As we anticipated considerable between‐study heterogeneity, the data were pooled using a random effects model (Hartung‐Knapp‐Sidik‐Jonkman). *I* ^2^ was reported as a measure for heterogeneity across studies. As influencer outlier analysis, a Baujat plot and leave‐one‐out analysis were performed. Potential publication bias was examined in a funnel plot and Egger's test.

## 3 Results

### 3.1 Neutrophil Functional Changes in CKD: A Systematic Review

Using defined search and eligibility criteria (Table S1), we identified 524 articles examining neutrophil function in CKD. After exclusion of duplicates, non‐English, and non‐original publications, and after an initial screening based on title and abstract, the full text of 238 articles was assessed. Of these, 146 were excluded because they only measured neutrophil‐to‐lymphocyte ratio or neutrophil numbers (*n* = 74), used patient material other than blood (*n* = 30), had no healthy control group (*n* = 41) or used a non‐adult control group vs. an adult patient group (*n* = 1). Thus, 92 studies were included in the systematic review (Figure 1). The most studied neutrophil response was ROS generation (*n* = 25), followed by phagocytosis (*n* = 19), surface marker expression (*n* = 14), apoptosis (*n* = 9), degranulation (*n* = 8) and NET formation (*n* = 4).

**FIGURE 2:**
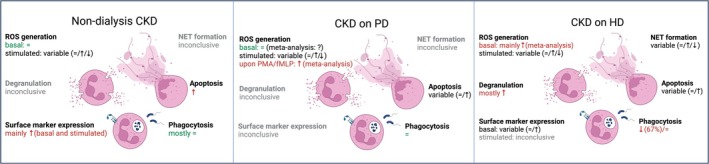
Summary of studies reporting on neutrophil parameters in specific CKD patient cohorts (compared to healthy controls). Graphical summary of Table 2. “Inconclusive” indicates no overall conclusion due to insufficient study number. CKD, chronic kidney disease; HD, hemodialysis; NET, neutrophil extracellular traps; n.s., not specified; PD, peritoneal dialysis; ROS, reactive oxygen species.

Figure 2 and Table 1 visually summarize the overall findings of the systematic review. Table S2 classifies the individual studies according to their effect (increase, decrease, unaltered) on each of the neutrophil readouts, whereas Tables S3–S8 provide full study details for each of the neutrophil readouts. A more detailed results description with additional details on the individual findings is provided in the Supplementary Results.

**TABLE 1:** Summary of studies reporting on neutrophil parameters in specific CKD patient cohorts (compared to healthy controls).

Neutrophil response	Non‐dialysis CKD			Peritoneal dialysis			Hemodialysis			CKD: mixed^d^ or n.s.	
Phagocytosis	4/6		2/6	5/5			5/15	10/15		1/2	1/2
Apoptosis (basal)	5/5			2/3		1/3	5/8		3/8	—	
Surface activation markers^a^ (basal)	4/5		1/5	—			4/8		4/8	1/1	
Surface activation markers^a^ (stimulated)	2/2 (LPS, fMLP)			—			1/1 (IL8)			1/1	
Degranulation (plasma markers)^b^	1/1			—			5/5			1/1	
Degranulation (neutrophil readouts)^b^	—			—			1/4	3/4		—	
NET formation (basal)	—			—			1/3	2/3		1/1	
NET formation (stimulated)	—			—			1/3	1/3	1/3	1/1	
ROS production (basal)	4/4			2/2			8/12	3/12	1/12	—	
ROS production (stimulation)^c^	3/7	2/7	3/7	3/5	2/5	1/5	7/17	2/17	9/17	—	

Increased
Unaltered
Reduced

*Note:* The colors indicate whether the reported neutrophil response in specific CKD patient cohorts is increased, unaltered or reduced compared to healthy controls. (orange = increased, grey = unaltered, blue = reduced).

Abbreviations: CKD, chronic kidney disease; NET, neutrophil extracellular traps; n.s., not specified; ROS, reactive oxygen species.

^a^ With inclusion of only CD11b, CD18, CD66b, and CD88 (Counted as increased if at least one of these was increased).

^b^ Counted as increased if at least one of the markers was increased.

^c^ Counted as increased (decreased) if at least with one of the stimuli, increased (decreased) ROS was observed in patients vs. controls. Thus, for a study with both an increased and decreased result depending on the stimulus, the study was counted in for both increased and decreased effects. Studies were counted as “unaltered” only if none of the tested conditions showed an increase or decrease in patients vs. controls.

^d^ Mixed non‐dialysis CKD, peritoneal dialysis, hemodialysis.

### 3.2 Phagocytosis

Neutrophil phagocytic capacity was mostly found unaltered in non‐dialysis CKD patients (unaltered: *n* = 4 studies [29, 34, 35, 36]; vs. reduced: *n* = 2 studies [27, 37]) and in CKD patients on PD (unaltered: *n* = 5 [27, 29, 34, 35, 37]). Instead, it was reduced in 67% (10 of 15) of studies in dialysis patients on HD (reduced: *n* = 10 [29, 30, 34, 35, 36, 38, 39, 40, 41, 42] vs. unaltered: *n* = 5 [25, 43, 44, 45, 46]) (Figure 2, Tables 1, S2 and S3).

### 3.3 Apoptosis

Neutrophil apoptosis was increased consistently in non‐dialysis CKD patients [13, 29, 47, 48, 49], but only in a subset of studies in CKD patients on HD or PD (increased in HD [13, 49, 50] or increased in PD [13], vs. unaltered in HD [20, 26, 29, 48, 51] or PD [29, 48]; Figure 2, Tables 1, S2 and S4).

### 3.4 Surface Activation Markers

CD11b, CD18, CD66b, and CD88 were the most frequently studied activation markers on neutrophils in CKD. Their analysis revealed generally increased neutrophil activation in non‐dialysis CKD patients in both basal conditions (increased: *n* = 4 [49, 52, 53, 54] vs. reduced: *n* = 1 [55]) as well as in conditions of stimulation (increased: *n* = 2 [53, 54]). In contrast, neutrophils from HD patients showed in basal conditions only in half of the studies an increase in (at least some of) these four activation markers (increased: *n* = 4 [18, 30, 33, 49]; unaltered: *n* = 4 [22, 52, 56, 57]). Insufficient data are available on stimulated neutrophils in HD patients (*n* = 1: unaltered [56]) as well as on neutrophils from PD patients (Figure 2, Tables 1, S2 and S5).

### 3.5 Degranulation

Degranulation refers to the active release of granule contents in response to neutrophil stimulation. In addition to (i) an increased MPO or neutrophil elastase release upon neutrophil stimulation, we also included in this chapter the following readouts reported in the identified studies: (ii) reduced basal levels of MPO or neutrophil elastase in neutrophils, and (iii) increased levels of neutrophil elastase, MPO, lactoferrin, or histone‐DNA in blood. All studies observed an increase in at least one of these readouts in either blood or isolated neutrophils in CKD patients compared to healthy controls (*n* = 8: 7× HD patients; 1× unspecified CKD stage) [24, 30, 58, 59, 60, 61, 62, 63]. Only one study compared both non‐dialysis CKD and HD patients to healthy controls, revealing increased plasma MPO levels only in HD patients [60] (Figure 2, Tables 1, S2 and S6).

### 3.6 NET Formation

Overall, highly variable data were observed for NET formation by neutrophils from HD patients (basal NET formation: increased: *n* = 2 [22, 64] vs. unaltered: *n* = 1 [30]; stimulated NET formation: increased: *n* = 1 [64]; decreased: *n* = 1 [30]; unaltered: *n* = 1 [22, 65]; Figure 2, Tables 1, S2 and S7). No comparison was possible to PD patients or non‐dialysis CKD patients due to a lack of data.

### 3.7 ROS Production

In basal, unstimulated conditions, an increased ROS production was observed in 8 of 12 studies on HD patients, whereas no changes were detected in non‐dialysis CKD patients (*n* = 4) or PD patients (*n* = 2). In conditions of neutrophil stimulation, findings on ROS production were highly variable in both non‐dialysis as well as PD and HD patients (Figure 2, Tables 1, S2 and S8). Neutrophils produce ROS mainly via the NADPH oxidase 2 (NOX2) at the plasma membrane or in intracellular compartments, producing extracellular and/or intracellular ROS [14]. Furthermore, different stimuli have been used to analyze neutrophil ROS production. Since this may add to the observed data variability, we subsequently performed a meta‐analysis of neutrophil ROS production with subgroup analyses.

In summary, the systematic review identified primarily reduced phagocytosis capacity but increased neutrophil degranulation and basal ROS production in neutrophils from CKD patients on hemodialysis compared to controls (Figure 2). Instead, phagocytosis and basal ROS production were mainly unaltered in non‐dialysis‐dependent CKD patients and CKD patients on peritoneal dialysis (Figure 2).

### 3.8 Neutrophil ROS Production in CKD: A Meta‐Analysis

With ROS production being the most studied neutrophil readout and an important contributor to CVD pathophysiology, we performed a meta‐analysis on ROS generation to summarize the available data and enable subgroup analyses to identify potential factors contributing to data heterogeneity in current literature. Since only eight studies investigated non‐dialysis dependent CKD patients, the meta‐analysis focused on ROS in dialysis patients (*n* = 24 studies, with patients either on HD or PD). A total of 18 studies used comparable methods for ROS measurements and provided sufficient details on datasets for inclusion in the meta‐analysis (Figure 1, Table 2). The other studies had to be excluded based on incomparable assay use (*n* = 4; assays: oxygen consumption by Clark electrode, *C. albicans* as only stimulus, C5a and FLPEP as only stimulus, experimental setup unclear), insufficient data sets (*n* = 2: number of controls unclear, results only shown as median) and patients reported to be only on newly initiated dialysis (*n* = 1) (Figure 1, Table S1).

**TABLE 2:** Characteristics of studies analyzing neutrophil ROS production in dialysis patients, as included in the meta‐analysis.

Study	Patient cohort	Neutrophils in full blood/isolated neutrophils	ROS Assay (I, intracellular) (E, extracellular) (T, total)	Stimulus	Overall study conclusion on ROS response in patients compared to healthy controls	Dialysis procedure
					(Basal/Stimulated)	
Cohen et al. [17]	HD	Full blood analysis	DHR oxidation (FC) (I)	*E. coli* & PMA	Decreased (stimulated)	High‐flux membranes (nitrocellulose triacetate)
Cohen‐Mazor et al. [18]	HD	PMNL	SOD‐inhibitable cytochrome c reduction (E)	PMA	Increased (stimulated)	Low‐flux polysulfone membranes
Cohen‐Hagai et al. [19]	HD	Full blood analysis	DHR oxidation (FC) (I)	*E. coli* & PMA	Decreased (stimulated)	n.s.
Guo et al. [20]	HD	PMNL	DCFH‐DA oxidation (FC) (I)	PMA, fMLP & *S. aureus*	Increased (unstimulated + stimulated with *S. aureus* and fMLP), unaltered (stimulated with PMA)	n.s.
Haynes et al. [21]	PD	PMNL	Lucigenin‐enhanced chemiluminescence (E)	fMLP	Increased (stimulated)	—
Kim et al. [22]	HD	PMNL	DCFH‐DA oxidation (plate reader) (T)	Unstimulated	Increased (unstimulated)	Standard bicarbonate dialysis & semisynthetic membranes
Kristal et al. [23]	HD	PMNL	SOD‐inhibitable cytochrome c reduction (E)	PMA	Increased (stimulated)	Polysulfone or cellulose triacetate dialysis membranes
Otaki et al. [24]	HD	PMNL	DCFH‐DA oxidation (FC) (I)	Unstimulated	Unaltered (unstimulated)	Either cellulose triacetate, excebrane, or polymethylmethacrylate membranes and bicarbonate dialysate
Patruta et al. [25]	HD	PMNL	SOD‐inhibitable cytochrome c reduction (E)	PMA	Increased (unstimulated) Decreased (stimulated)	Bicarbonate hemodialysis using biocompatible membrane material made of polysulfone or cellulose triacetate
Perianayagam et al. [26]	HD	Full blood analysis	DCFH‐DA oxidation (FC) (I)	Unstimulated	Increased (unstimulated)	High‐flux dialyzers
Porter et al. [27]	PD	PMNL	Luminol‐enhanced chemiluminescence (T); DCFH‐DA oxidation (FC) (I)	*S. epidermidis*	Unaltered (stimulated)	—
Rao et al. [28]	HD	PMNL	SOD‐inhibitable cytochrome c reduction (E)	PMA, fMLP	Decreased (stimulated PMA), unaltered (stimulated fMLP)	Polysulphone (PS), cuprophane (CU), cellulose (acetate, triacetate) (CA/CT)
Sardenberg et al. [29]	HD PD	Full blood analysis	DCFH‐DA oxidation (FC) (I)	Unstimulated, *S. aureus*, PMA, fMLP	Increased (stimulated with bacterial and fMLP+basal for CAPD), Unaltered (stimulated + unstimulated HD, stimulated with PMA for CAPD)	HD: polysulfone dialyzers CAPD: exchanges of glucose, lactate‐based solutions that usually included one 4.25% glucose solution exchange
Sela et al. [13]	PD HD	PMNL	SOD‐inhibitable cytochrome c reduction (E)	unstimulated, PMA, Zymosan	Increased (stimulated PMA), Decreased (stimulated Zymosan)	CAPD: 8 L/d in four exchanges (three isotonic 1.36% and one hypertonic 3.86% glucose solutions); HD: low‐flux polysulfone membranes with bicarbonate dialysate
Talal et al. [30]	HD	Neutrophils	Extracellular ROS with chemilumescence (E)	PMA, LPS	Decreased (stimulated)	n.s.
Ward et al. [31]	HD	PMNL	Cytochrome c reduction (E)	TNF, fMLP	Unaltered (unstimulated), Decreased (stimulated)	Reused dialyzers containing cellulose acetate membrane
Ward et al. [32]	HD	Full blood analysis	DCFH‐DA oxidation (FC) (I)	Unstimulated, *S. aureus*	Increased (unstimulated + stimulated)	High‐flux dialyzers containing polysulfone and cellulose triacetate membranes
Yoon et al. [33]	HD	Full blood analysis	DHE oxidation, DHR oxidation (FC) (I)	Unstimulated	Increased (unstimulated)	Cellulose acetate dialyzers

*Note:* For ROS location: (I) intracellular; (E) extracellular; (T) total = intra‐ and extracellular. All studies were observational studies.

Abbreviations: DCFH‐DA, 2′,7′‐dichlorodihydrofluorescein diacetate; DHE, dihydroethidium; DHR, dihydrorhodamine; EDTA, ethylenediaminetetraacetic acid; FC, flow cytometry; HD, hemodialysis; MFI, mean fluorescence intensity; n.s., not specified; obs., observational; PD, peritoneal dialysis; PMNL, polymorphonuclear leukocyte.

No publication bias was detected (Funnel plot in Figure S1; Eggers' test for Funnel plot asymmetry: *p* = 0.089). Influential outlier analyses revealed Cohen‐Hagai et al. [19] as well as Kim et al. [22] as studies impacting most on the overall heterogeneity of the meta‐analysis (Figure S2A). However, since these studies were not identified as unique strong influencers of the pooled result in both performed influential outlier tests (Figure S2A,B), no studies were excluded from the meta‐analysis. Overall, the meta‐analysis showed no significant difference in ROS production between neutrophils of dialysis patients and healthy controls, neither on overall level (Hedges *g* = 0.06; 95% CI: [−0.54; 0.66]) nor when analyzing specifically intra‐ or extracellular ROS (Figure S3, intracellular ROS: Hedges *g* = −0.08; 95% CI: [−0.95; 0.80]; extracellular ROS: Hedges *g* = 0.07; 95% CI: [−0.73; 0.88]).

**FIGURE 3:**
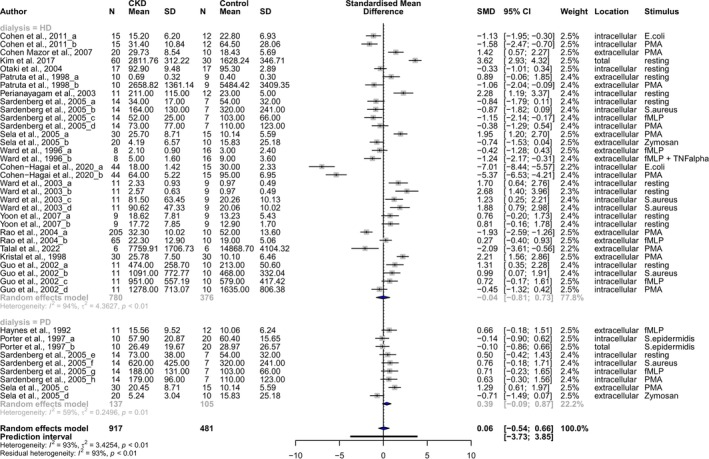
Meta‐analysis shows no overall significant difference in neutrophil ROS production between CKD patients on either hemodialysis or peritoneal dialysis versus controls. Forest plot of neutrophil ROS production in dialysis patients compared to healthy controls, with subgroup analysis of the effect of dialysis type. CI, confidence interval; CKD, chronic kidney disease; *E. coli* , *Escherichia coli* ; HD, hemodialysis; fMLP, N‐formyl‐methionyl‐leucyl‐phenylalanine; PD, peritoneal dialysis; PMA, Phorbol 12‐myristate 13‐acetate; *S. aureus* , *Staphylococcus aureus* ; SD, standard deviation; *S. epidermidis* , *Streptococcus epidermidis*; TNFα, tumor necrosis factor α.

However, the meta‐analysis revealed an overall high data heterogeneity (*I* ^2^ = 93%) and highlighted that even within the same study conflicting results could be detected dependent on the type of dialysis performed [29] (i.e., HD vs. PD), whether resting vs. stimulated neutrophils were examined [25] or dependent on which stimulus was applied [13, 20, 28, 31]. A second meta‐analysis with subgrouping of studies based on the type of dialysis could not reveal an overall significant difference in neutrophil ROS generation in either HD or PD patients compared to healthy controls (HD: Hedges *g* = −0.04, 95% CI: [−0.81; 0.73]; PD: Hedges *g* = 0.39, 95% CI: [−0.09; 0.87], Figure 3), but again highlighted the presence and type of stimulus as an important influencing factor.

**FIGURE 4:**
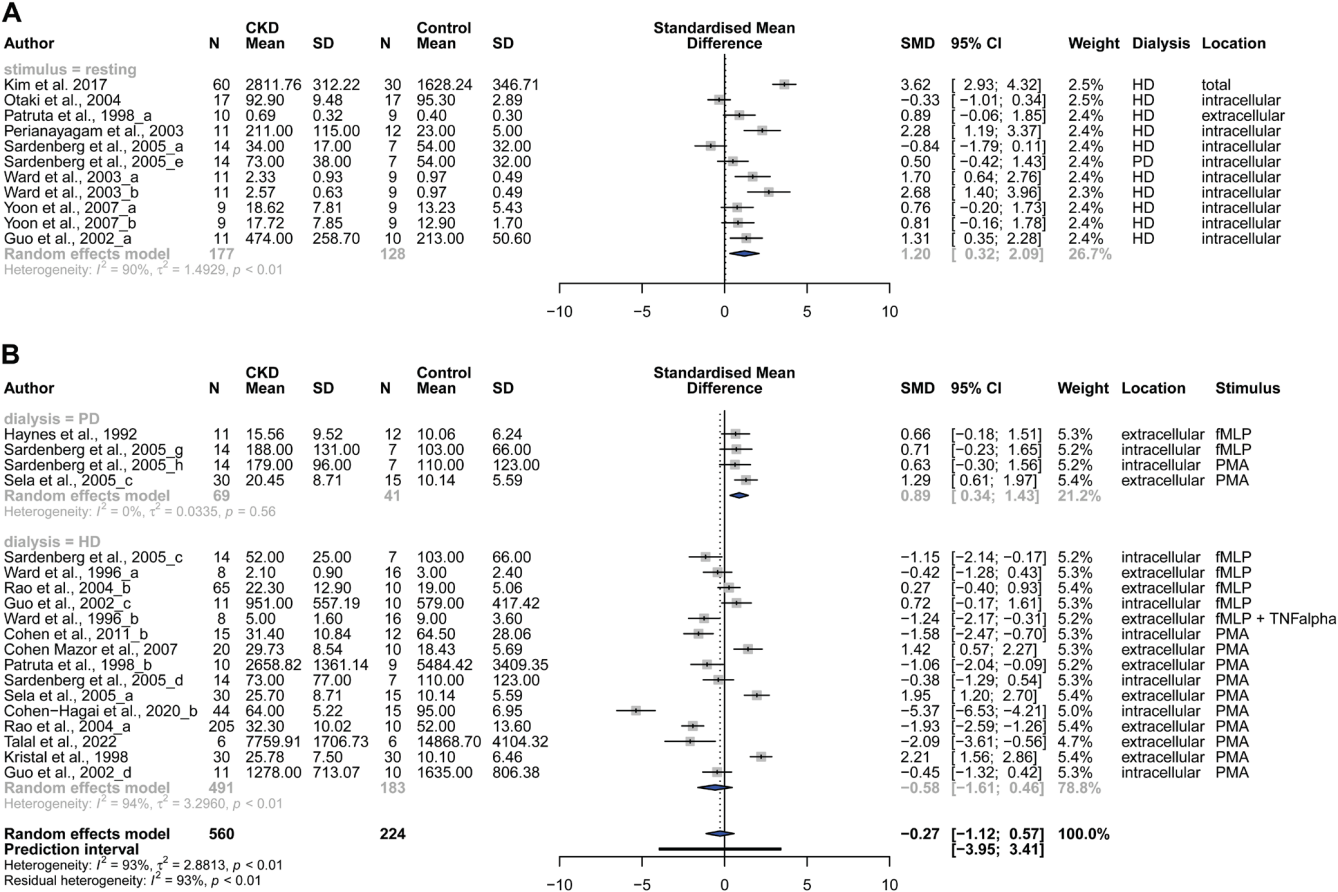
Meta‐analysis of ROS production by resting or stimulated neutrophils from CKD patients on dialysis. (A) Meta‐analysis shows increased ROS production by resting neutrophils from predominantly hemodialysis‐patients versus controls. Forest plot of neutrophil ROS production in dialysis patients compared to healthy controls, with analysis of the subgroup “resting neutrophils”. Provided is a zoomed extract from the full meta‐analysis of the effect of stimulus presence and type on ROS generation in neutrophils, as displayed in Figure S4. (B) Meta‐analysis shows increased ROS generation in neutrophils of PD patients, when stimulated with stimuli reflecting sterile inflammatory conditions. Forest plot of neutrophil ROS production in dialysis patients compared to healthy controls upon neutrophil stimulation with fMLP, fMLP and TNFα or PMA. CI, confidence interval; CKD, chronic kidney disease; fMLP, N‐formyl‐methionyl‐leucyl‐phenylalanine; HD, hemodialysis; PD, peritoneal dialysis; PMA, Phorbol 12‐myristate 13‐acetate; SD, standard deviation, TNFα, tumor necrosis factor α.

We therefore subdivided the included studies depending on the absence vs. presence as well as the type of stimulus. This revealed a significantly increased ROS production in resting neutrophils from dialysis patients (of which 91% HD patients) vs. healthy controls (Figure 4A; resting: Hedges *g* = 1.20, 95% CI: [0.32; 2.09]). Upon neutrophil stimulation, data remained highly variable (Figure S4). Stimuli included both direct bacterial or fungal triggers as well as stimuli that may rather reflect sterile inflammatory conditions, being PMA and fMLP (officially called fMLF, a prototypical formyl peptide used in research, with similar formylated peptides produced by mitochondria and released upon cell injury [66]). When focusing specifically on these latter, neutrophils from PD patients showed an overall increased ROS production compared to healthy controls (Figure 4B; PD: Hedges *g* = 0.89, 95% CI: [0.34; 1.43], *I* ^2^ = 0%). In contrast, no significant effect could be observed in HD patients: ROS production upon neutrophil stimulation with PMA, fMLP, or fMLP following prior TNFα stimulation was identified to be significantly decreased in 7 out of 15 datasets on HD patients, though unaltered (*n* = 5 datasets) or increased (*n* = 3 datasets) in the remaining 8 datasets. (Figure 4B; HD: Hedges *g* = −0.58; 95% CI: [−1.61; 0.46], *I* ^2^ = 94%).

All combined, these meta‐analyses indicate that in basal, unstimulated conditions, ROS production is increased in neutrophils from CKD patients on HD, while CKD patients on PD are insufficiently investigated. Upon sterile inflammatory stimulation of neutrophils, ROS production is increased in CKD patients receiving PD, with findings yet inconclusive for CKD patients on HD due to high data variability (Figure 2).

## 4 Discussion

Our systematic review revealed that neutrophils from CKD patients on HD generally show reduced phagocytosis capacity (reflecting a reduced response to an acute infection) but increased neutrophil degranulation and basal ROS production (reflecting an increased basal inflammatory response) compared to healthy controls. In contrast, neutrophils from non‐dialysis‐dependent CKD patients and CKD patients on PD mostly showed unaltered phagocytosis and basal ROS production (Figure 2). A meta‐analysis confirmed an increased basal ROS production in neutrophils of CKD patients on HD. Upon neutrophil stimulation with sterile inflammatory triggers, ROS production was also increased in neutrophils from PD patients.

Increased basal ROS production in resting neutrophils from HD patients points to enhanced basal cellular priming, and the procedure of HD can indeed be expected to affect neutrophil phenotype more than in the case of PD. Contributing factors include cellular stress induced by the HD procedure during which neutrophils are regularly exposed to shear stress and exogenous materials (dialysis membrane, (heparinized) plastic tubing, etc.) for a longer time. This is absent in non‐dialysis CKD patients, but also in PD patients in whom the peritoneum functions as an endogenous dialysis membrane. Other causes of HD‐induced cellular stress could be uremic retention solute accumulation in between HD procedures (with higher fluctuation in circulating levels compared to the continuous PD procedure), complement activation, chronic low‐grade inflammation [67, 68] or iron overload [20, 24], amongst others. The high variability in neutrophil readouts (including also apoptosis, surface activation markers, NET formation and phagocytosis) observed in HD patients may be due to differences in dialysis membranes [40] and modalities used, which may trigger alterations in uremic retention solute removal or dialysis‐induced inflammatory reactions and cellular stress. In line, a transient increase in neutrophil activation markers during the HD procedure has been observed dependent on the dialysis membrane used [52].

Neutrophil priming and increased basal ROS levels could also explain a secondary functional impairment with a reduced fold‐change upregulation of ROS production upon neutrophil stimulation (which has been described for NET formation in HD patient‐derived neutrophils [22]) or even reduced ROS production by stimulated neutrophils in HD patients [31] (Table 1, Figure 4B). This supports the hypothesis that neutrophils of HD patients may already be in a primed state due to the uremic and/or inflammatory milieu they are consistently exposed to, an effect that could be mimicked when incubating neutrophils from healthy individuals in plasma from HD patients [31].

On the other hand, basal cellular priming can also trigger increased secondary responses (“positive priming”), with multiple studies on neutrophils in HD patients also reporting on increased stimulation‐induced ROS production (Table 1, Figure 4B). However, in these studies, the results were dependent on the stimulus used (Figure 4A, Table S8) [13, 20, 28, 31]. For example, Sela et al. found an increase in ROS production upon stimulation of HD patient‐derived neutrophils with PMA (mimicking sterile inflammation) but a decrease with Zymosan (a component of the cell wall of yeast, mimicking microbial infection) [13]. The latter may be linked to the high susceptibility of CKD patients to acute infection [69, 70].

The overall high variability in findings on neutrophil activity readouts in CKD between different studies could be due to a high patient population heterogeneity with regard to CKD stage, primary underlying CKD pathology, existing comorbidities such as hypertension, obesity, diabetes, and CVD [8], as well as medication. For example, diabetes—a known cause and promoter of inflammation and oxidative stress [71], neutrophil activation [72] as well as of CKD [8]—was an exclusion criterium in only 17% of the included studies and could well be a variable factor between different studies of CKD patients. Towards the use of immunosuppressants, six of the 18 studies included in the meta‐analysis explicitly excluded patients on immunosuppressive therapy, and another two excluded patients with immunological disorders or with diseases associated with neutrophil dysfunction. Moreover, the dialysis type, duration on dialysis [73], and for HD patients also the timing of neutrophil analysis in relation to dialysis application and the applied dialysis membrane (which varied throughout the studies included in the meta‐analysis) may contribute to the high interstudy data heterogeneity in the meta‐analysis.

Also, the methods for analyzing ROS levels in neutrophils varied throughout the included papers, both in terms of analyzing extracellular vs. intracellular ROS (or a combination of both), as well as in terms of the specific assay used and ROS type measured. Neutrophils mainly produce ROS via the NADPH oxidase (NOX2), which reduces oxygen to the superoxide radical (O_2_^•‐^). Superoxide can either be produced at the plasma membrane (extracellular ROS) or in intracellular compartments (intracellular ROS) [14]. Superoxide rapidly dismutates to hydrogen peroxide (H_2_O_2_) by superoxide dismutase (SOD) or spontaneously, and can also convert to other types of ROS (e.g., peroxynitrite (ONOO^−^)). PMA is a potent inducer of both intra‐ and extracellular ROS production. The produced ROS is subsequently important for neutrophil function. For example, NADPH‐oxidase‐dependent intracellular ROS production is important for the killing capacity of neutrophils towards microbes, as well as for PMA‐induced NETosis [14]. In the meta‐analysis, 9 of 18 studies are based on the detection of superoxide. With superoxide rapidly converting to peroxynitrite or H_2_O_2_, measuring superoxide is a more unreliable ROS detection method compared to H_2_O_2_, which is less reactive. Overall, the use of chemical probes to measure ROS has several pitfalls, as extensively discussed before [74, 75]. Briefly, lack of specificity, no subcellular localization, indirect mechanism of oxidation, photo‐oxidation, and the fact that these probes are prone to artifacts are amongst the main problems (Table S9). For example, probes such as dihydrorhodamine also react with reactive nitrogen species. It is also important to mention that ROS production and removal are highly compartmentalized reactions and are unlikely to have effects far from the site where they are generated [76]. Understanding these limitations helps to interpret the great variability in some of the conditions discussed above. Of note, a lack of ROS changes in some conditions does not necessarily mean that ROS do not play an important role in CKD. It would be interesting to analyze specific ROS at defined cellular compartments to understand the causal role of different redox systems in CKD (e.g., mitochondria versus NADPH oxidase). Finally, ROS are important signaling molecules; therefore, understanding what the “optimal” ROS levels for cellular function are and when this threshold is shifted towards pathophysiology is still a highly active field of research [77].

Circulating blood neutrophils exhibit a remarkable heterogeneity. At any given time, various phenotypes of neutrophils coexist, with young vs. aged neutrophils representing a classic example of phenotypic heterogeneity at steady state. In disease states, such heterogeneity becomes more complex as discrete phenotypes of activated, primed, and immature neutrophils appear [78, 79]. Yet, these states are not just phenotypes discernable by surface markers, but indeed exhibit differential responses in terms of NET release, phagocytosis, adhesion, and ROS production. Thus, analyses based on assessing neutrophil functionality on a bulk scale may not suffice to identify altered responsiveness of neutrophil phenotypes. As an example, ROS production is often assessed in plate reader assays where a signal is recorded over time and possibly normalized by the number of cells seeded. Such population‐based analysis disregards differential responses of subpopulations, information only single cell‐based assays can reveal. Thus, flow cytometry‐based analysis of ROS production in different activation and maturation stages of neutrophils would yield more robust and detailed information of neutrophil responses. In addition to subset‐specific responses, methods of neutrophil extraction have tremendous effects on neutrophil responses. Lysis of red blood cells, density gradient centrifugation, and temperature changes all impact the phenotypic appearance of neutrophils and functionality, and different isolation protocols across studies assessed here may at least in part explain data heterogeneity. Moreover, the isolation protocol may even get rid of certain neutrophil subsets. As an example, low‐density granulocytes are found in the PBMC fraction rather than in the neutrophil fraction in density gradient‐based isolation procedures. Finally, and in addition to what has been described above for ROS production, it is key to provide details on experimental procedures. Phagocytosis assays, for example, are often performed with bacteria‐based bioparticles. As a considerable number of these bioparticles are only bound to neutrophil surfaces and not internalized, newer versions of these bioparticles are conjugated with a pH‐sensitive dye allowing for the recording of the acidification in the phagolysosome. Yet, in the body, bacteria are always opsonized either with antibodies or with activated complement, reagents often not used in ex vivo phagocytosis assays. Thus, the use of different opsonins and consequently different phagocytosis pathways may explain part of the data heterogeneity observed here.

A limitation of our study is the large time span between the included studies, ranging from 1990 until 2023. This may have impacted disease management (and thus characteristics of patients and their neutrophils), dialysis methods as well as research methods (e.g., differences in neutrophil isolation, different method of ROS analysis). Furthermore, non‐dialysis dependent CKD patients were not further specified according to CKD stage. Finally, we classified the presence of different neutrophil granule markers and histone‐DNA in blood as parameters for “degranulation”. However, these are not necessarily related to degranulation or even secretion but could also result from neutrophil death or from other cell types (as e.g., MPO from monocytes).

## 5 Conclusions

In conclusion, our study identified reduced phagocytic capacity but increased degranulation and basal ROS production in neutrophils from CKD patients on HD. This reflects a chronic inflammatory response but reduced responsiveness of neutrophils to an acute infection. Instead, increased basal ROS and reduced phagocytosis were mostly not detected in CKD patients on PD or non‐dialysis‐dependent CKD patients. Nonetheless, caution remains required since in general only a limited number of studies have addressed these patient groups, and additional studies on surface markers, NET formation, and degranulation markers are needed. A more detailed and side‐by‐side comparison of patients in stages CKD2–4 for different readouts of neutrophil function as well as studying neutrophil function in patients with CKD in relation to comorbidities (as diabetes) may provide further insight into the inherent effect of CKD on neutrophil activity.

We suggest future studies assessing the detailed neutrophil phenotype to combine the analysis of different activation markers on the neutrophil surface in whole blood, a fluorescent‐based phagocytosis bead assay as well as an assay for NET formation on isolated neutrophils. Analysis is recommended in both basal conditions as well as upon activation with different stimuli, such as PMA vs. IL8 or fMLP, since different signaling mechanisms may be differentially affected by the underlying pathology. Although PMA is a frequently applied stimulus, it is a synthetic stimulus that directly activates protein kinase C without the aid of any membrane receptor. When analyzing ROS, a flow cytometry‐based assay has the advantage to focus on neutrophils in a whole blood setting and to analyze the readout per cell. Analyzing mitochondrial versus NADPH‐oxidase would be interesting, and given the inherent difficulties with ROS assays described above, a combination of different assays with appropriate controls is advisable. Depending on the research question, exclusion of patients on immunosuppressive therapy is recommended, and patient characteristics and clinical details (including age, sex, CKD stage, comorbidities and medication, time on dialysis and time of blood sampling relative to the dialysis sessions) should be reported.

To our knowledge, no specific outcome studies investigating the association of markers of increased neutrophil activity to cardiovascular outcome have been performed in CKD patients so far, although this would be of high clinical relevance. Patients with CKD have an increased cardiovascular risk, to which increased neutrophil activation, ROS production, and NET formation [10] but also altered neutrophil interactions with endothelium [80] and platelets may contribute. Thus, a further clarification of the impact of CKD on these neutrophil responses also in CKD2‐4 could support future management of chronic inflammation and cardiovascular risk in CKD patients.

## Author Contributions

**Jane Sophie Lauxen:** investigation, data curation, formal analysis, visualization, writing – original draft, writing – review and editing. **Sonja Vondenhoff:** validation, writing – review and editing. **Carolina Victoria Cruz Junho:** investigation, validation, writing – review and editing. **Philipp Martin:** validation, writing – review and editing. **Susanne Fleig:** writing – review and editing. **Katharina Schütt:** writing – review and editing. **Ulrike Schulze‐Späte:** writing – review and editing. **Oliver Soehnlein:** writing – review and editing. **Leticia Prates‐Roma:** writing – review and editing. **Yvonne Döring:** writing – review and editing. **Constance C. F. M. J. Baaten:** methodology, software, visualization, writing – review and editing. **Heidi Noels:** conceptualization, funding acquisition, methodology, supervision, validation, writing – review and editing.

## Conflicts of Interest

The authors declare no conflicts of interest.

## Supporting information


**Data S1.** supporting Information.

## Data Availability

Data extracted from the included studies and used for all analyses are presented in detail in Table 2 and the meta‐analysis figures. The extracted data values used in the meta‐analysis are also publicly available as Supplemental Data Set via the University library of the RWTH Aachen (DOI: https://doi.org/10.18154/RWTH‐2025‐03170).
