# Supplementary material for: Neutrophil Function in Patients With Chronic Kidney Disease: A Systematic Review and Meta‐Analysis
Source: Acta Physiol (Oxf). 2025 May 24;241(6):e70057. doi: 10.1111/apha.70057 (PMC12102643; doi:10.1111/apha.70057)
Supplement: Supplementary file 1 — Data S1. supporting Information. [file APHA-241-e70057-s001.pdf]

Neutrophil function in patients with chronic kidney disease: a systematic review and meta-analysis

By Lauxen et al.

Table of contents

Supplementary results with references	p. 2
Supplementary tables	p. 10
Supplementary figures	p. 20

Supplementary results

Neutrophil functional changes in CKD: a systematic review

Phagocytosis. Results on phagocytosis activity in CKD were highly variable, also within the same study (n=19 studies; n=13 decreased[1-13]; n=13 unaltered[3,4,6,10-19] in CKD vs. healthy controls; **Suppl. Table 2**). The results were partly dependent on whether CKD patients were on dialysis as well as on the dialysis type: an unaltered phagocytic capacity was reported in four of six studies on non-dialysis CKD patients (n=4 unaltered[3,4,6,12]; vs. n=2 reduced phagocytosis[10,13]) and in all studies on PD patients (n=5[3,4,6,10,13]) (**Table 1**). In contrast, of the 15 studies analyzing HD patients, 67% (10 of 15) detected reduced phagocytosis capacity vs. controls[1-8,11,12] and one study detected reduced or unaltered phagocytosis dependent on the dialysis membrane used[11], with the other five studies not identifying differences[14-17,19] (**Table 1; Suppl. Table 3**). A similar conclusion holds when only accounting the studies that simultaneously compared non-dialysis and dialysis patients (HD or PD) to controls (**Suppl. Table 3**). In conclusion, neutrophil phagocytic capacity was mostly found unaltered in non-dialysis CKD patients and CKD patients on PD, whereas it was reduced in 67% of studies in dialysis patients on HD.

Apoptosis. Also findings on neutrophil apoptosis were partly dependent on whether non-dialysis CKD patients or patients on dialysis were analyzed: All five studies analyzing neutrophils of non-dialysis patients reported increased apoptosis compared to healthy controls[4,20-23]. Instead, of the eight studies analyzing neutrophils of dialysis patients, only three reported on increased apoptosis in HD[22-24] or PD patients[22], whereas the other (n=5) did not detect differences in either HD[4,21,25-27] or PD patients[4,21] vs. controls (**Table 1**, **Suppl. Tables 2 and 4**). Summarized, neutrophil apoptosis was increased consistently in non-dialysis CKD patients, but only in subset of studies in CKD patients on HD or PD.

Surface activation markers. *(Basal)* In unstimulated conditions, 10 of 14 studies (71%) reported an increased surface activation marker expression in CKD patients vs. healthy

controls (e.g. for CD11b and CD18)[8,23,28-35] - although not always of all investigated markers – and/or a reduction of surface CD62L, which is known to be shed upon neutrophil activation[32,35] (**Suppl. Tables 2 and 5**). For CD11b, CD18, CD66b and CD88 as the most frequently studied activation markers, four of five studies analyzing non-dialysis CKD patients found increased expression compared to healthy controls (n=4[23,31,32,34]), while one found reduced expression (n=1[36]). In contrast, HD patients were reported to have one or more of these four activation markers increased only in 50% of the studies (increased: n=4[8,23,29,33]; unaltered: n=4[30,32,37,38]) (**Table 1, Suppl. Table 5B**). When comparing CD62L before vs. after a hemodialysis session, CD62L was reduced after dialysis, indicating neutrophil activation during the dialysis session[35]. One study identified a decreased surface expression of the chemokine receptor CXCR1 in dialysis patients compared to controls[38], which is reduced upon neutrophil activation. Two studies on HD patients could not detect any investigated activation marker (CD11b, TLR4/9, CD88) to be altered in basal conditions[37,39] (**Suppl. Table 5B**).

(Stimulated) Only four studies examined the impact of CKD on surface activation markers of stimulated neutrophils. Three studies (2x non-dialysis CKD; 1x not-specified CKD cohort) showed a stronger CD11b increase in CKD patients compared to healthy controls (e.g. upon stimulation with phorbol myristate acetate (PMA), N-formyl-methionyl-leucyl-phenylalanine (fMLP) or lipopolysaccharides (LPS))[28,34,40], although other markers as CD62L or CD11a remained unaltered (**Suppl. Table 5B**). One study in HD patients (IL8-stimulation) could not detect a difference in CD11b expression compared to healthy controls[37] (**Table 1**, **Suppl. Table 5B**).

In summary, analysis of surface activation markers revealed generally increased neutrophil activation in non-dialysis CKD patients in both basal and stimulated conditions, but only in around half of studies on unstimulated neutrophils from HD patients. Insufficient data are yet available on stimulated neutrophils in HD patients as well as on neutrophils from PD patients.

Degranulation. All studies observed an increase in at least one of the neutrophil degranulation readouts in either blood or isolated neutrophils in CKD patients compared to controls (n=8: 7x

HD patients; 1x unspecified CKD stage)[8,41-47]. These readouts included (i) increased levels of neutrophil elastase, MPO, lactoferrin or histone-DNA in blood, (ii) reduced basal levels of MPO or neutrophil elastase in neutrophils, or (iii) an increased MPO or neutrophil elastase release upon neutrophil stimulation (**Suppl. Tables 2 and 6**). Only one study compared non-dialysis CKD and HD patients, revealing increased plasma MPO levels only in HD patients[44] (**Suppl. Table 6**). In summary, neutrophil degranulation was mainly found to be increased in HD patients (**Figure 2)**, with no comparison possible to PD patients or non-dialysis CKD patients due to a lack of sufficient data.

NET formation. NET formation was predominantly assessed by quantification of nucleic acid by Sytox-Green-fluorescence in isolated neutrophils. Four studies analyzed NET formation in neutrophils from CKD patients (3x HD; 1x combining patients with HD, PD and non-dialysis CKD), however, with variable results (**Table 1, Suppl. Tables 2 and 7**): *(Basal)* Using resting neutrophils, two studies observed an increased NET formation vs. healthy controls[30,48] whereas the other two studies did not observe differences[8,49]. *(Stimulated)* Upon stimulation with PMA or calcium ionophore (A23187), two studies showed no alterations between dialysis-patients and controls[30,49], one study showed increased[48] and one decreased NET formation[8]. In summary, highly variable data were observed for NET formation by neutrophils from HD patients, with no comparison possible to PD patients or non-dialysis CKD patients due to a lack of data.

ROS production. Neutrophils produce ROS via the NADPH-oxidase (NOX2) at the plasma membrane (extracellular ROS) or in intracellular compartments (intracellular ROS)[50]. Findings on neutrophil ROS production in CKD patients varied strongly: *(Basal)* From 13 studies that measured basal ROS levels in neutrophils of CKD patients, seven found increased ROS compared to healthy controls[3,15,25,27,30,33,44], while seven found the parameter unaltered[3,4,18,43,44,51,52] with sometimes variable results obtained within the same study dependent on the patient cohort[3,44]; only one study found decreased basal ROS production in neutrophils from CKD patients[53] (**Suppl. Table 2**). When discriminating between non-dialysis CKD, PD and HD patients, an increased basal ROS production was observed in 8 of 12 studies

on HD patients, whereas no changes were detected in non-dialysis CKD patients (n=4) or PD patients (n=2) (**Table 1, suppl. Table 8**).

(Stimulated) A high data variability was also observed for ROS production in stimulated neutrophils of CKD patients (n=20 publications), even within the same study depending on the patient cohort or stimulus analyzed, with respectively nine, seven and ten studies observing decreased[8,15,22,53-58], unaltered[3,4,10,14,18,27,54] or increased[3,4,18,22,27,29,51,52,59,60] ROS production in CKD compared to healthy controls (**Suppl. Table 2**). Data remained highly variable even when discriminating between non-dialysis, PD and HD-patients (**Table 1, suppl. Table 8**). In non-dialysis CKD patients (n=7 publications), stimulated ROS production was observed to be increased[18,22,59], decreased[10,22,58] or unaltered[3,4,18,58] dependent on the stimulus analyzed (**Table 1**). Similarly, a high variability was seen in HD and PD patients (**Table 1**).

Overall, a high data variability was observed when summarizing neutrophil ROS production in CKD patients, urging a closer look into data variability and potential subgroups using a meta-analysis.

Supplemental References

1. Alexiewicz JM, Smogorzewski M, Klin M, Akmal M, Massry SG. Effect of treatment of hemodialysis patients with nifedipine on metabolism and function of polymorphonuclear leukocytes. *Am J Kidney Dis*. 1995;25:440-444. doi: 10.1016/0272-6386(95)90106-x
2. Cuffini AM, Tullio V, Giacchino F, Mandras N, Scalas D, Belardi P, Merlino C, Carlone NA. Impact of co-amoxiclav on polymorphonuclear granulocytes from chronic hemodialysis patients. *Am J Kidney Dis*. 2001;37:1253-1259. doi: 10.1053/ajkd.2001.24530
3. Lemesch S, Ribitsch W, Schilcher G, Spindelböck W, Hafner-Gießauf H, Marsche G, Pasterk L, Payerl D, Schmerböck B, Tawdrous M, et al. Mode of renal replacement therapy determines endotoxemia and neutrophil dysfunction in chronic kidney disease. *Sci Rep*. 2016;6:34534. doi: 10.1038/srep34534
4. Sardenberg C, Suassuna P, Andreoli MC, Watanabe R, Dalboni MA, Manfredi SR, dos Santos OP, Kallas EG, Draibe SA, Cendoroglo M. Effects of uraemia and dialysis modality on polymorphonuclear cell apoptosis and function. *Nephrol Dial Transplant*. 2006;21:160-165. doi: 10.1093/ndt/gfi095
5. Alexiewicz JM, Smogorzewski M, Fadda GZ, Massry SG. Impaired phagocytosis in dialysis patients: studies on mechanisms. *Am J Nephrol*. 1991;11:102-111. doi: 10.1159/000168284
6. Muniz-Junqueira MI, Braga Lopes C, Magalhães CA, Schleicher CC, Veiga JP. Acute and chronic influence of hemodialysis according to the membrane used on phagocytic function of neutrophils and monocytes and pro-inflammatory cytokines production in

chronic renal failure patients. *Life Sci*. 2005;77:3141-3155. doi: 10.1016/j.lfs.2005.03.034
7. Pappas EM, Mpournaka S, Katopodis P, Chardalias A, Tsakas S, Theodoros T, Evangelos E, Katopodis KP, Goumenos DS. The Effect of Dialysis Modality and Membrane Performance on Native Immunity in Dialysis Patients. *Pril (Makedon Akad Nauk Umet Odd Med Nauki)*. 2019;40:25-32. doi: 10.2478/prilozi-2019-0011
8. Talal S, Mona K, Karem A, Yaniv L, Reut HM, Ariel S, Moran AK, Harel E, Campisi-Pinto S, Mahmoud AA, et al. Neutrophil degranulation and severely impaired extracellular trap formation at the basis of susceptibility to infections of hemodialysis patients. *BMC Med*. 2022;20:364. doi: 10.1186/s12916-022-02564-1
9. Peng DZ, Liu XW, Huang L, Zhu XF, Zheng YQ, Wang LX. Relationship between leptin and chronic inflammatory state in uremic patients. *Eur Rev Med Pharmacol Sci*. 2014;18:2882-2885.
10. Porter CJ, Burden RP, Morgan AG, Daniels I, Fletcher J. Impaired bacterial killing and hydrogen peroxide production by polymorphonuclear neutrophils in end-stage renal failure. *Nephron*. 1997;77:479-481. doi: 10.1159/000190328
11. Banche G, Allizond V, Giacchino F, Mandras N, Roana J, Bonello F, Belardi P, Tullio V, Merlino C, Carlone N, et al. Effect of dialysis membrane biocompatibility on polymorphonuclear granulocyte activity in dialysis patients. *Nephrol Dial Transplant*. 2006;21:3532-3538. doi: 10.1093/ndt/gfl415
12. Mahajan S, Kalra OP, Asit KT, Ahuja G, Kalra V. Phagocytic polymorphonuclear function in patients with progressive uremia and the effect of acute hemodialysis. *Ren Fail*. 2005;27:357-360. doi: 10.1081/JDI-200065223
13. Porter CJ, Burden RP, Morgan AG, Daniels I, Fletcher J. Impaired polymorphonuclear neutrophil function in end-stage renal failure and its correction by continuous ambulatory peritoneal dialysis. *Nephron*. 1995;71:133-137. doi: 10.1159/000188700
14. Anding K, Gross P, Rost JM, Allgaier D, Jacobs E. The influence of uraemia and haemodialysis on neutrophil phagocytosis and antimicrobial killing. *Nephrol Dial Transplant*. 2003;18:2067-2073. doi: 10.1093/ndt/gfg330
15. Patruta SI, Edlinger R, Sunder-Plassmann G, Hörl WH. Neutrophil impairment associated with iron therapy in hemodialysis patients with functional iron deficiency. *J Am Soc Nephrol*. 1998;9:655-663. doi: 10.1681/asn.V94655
16. Scalas D, Banche G, Merlino C, Giacchino F, Allizond V, Garneri G, Patti R, Roana J, Mandras N, Tullio V, et al. Role of caspofungin in restoring the impaired phagocyte-dependent innate immunity towards Candida albicans in chronic haemodialysis patients. *Int J Antimicrob Agents*. 2012;39:73-76. doi: 10.1016/j.ijantimicag.2011.09.015
17. Tullio V, Cuffini AM, Giacchino F, Mandras N, Roana J, Comune L, Merlino C, Carlone NA. Combined action of fluconazole and PMNs from uremic patients in clearing intracellular Candida albicans. *J Chemother*. 2003;15:301-303. doi: 10.1179/joc.2003.15.3.301
18. Ward RA, McLeish KR. Polymorphonuclear leukocyte oxidative burst is enhanced in patients with chronic renal insufficiency. *J Am Soc Nephrol*. 1995;5:1697-1702. doi: 10.1681/asn.V591697
19. Schauer S, Stein G, Süss J, Falkenhagen D, Linss W. Phagocytosis activity of polymorphonuclear cells of normal persons and dialysis patients is influenced by different dialysis membranes. *Nephrol Dial Transplant*. 1991;6 Suppl 3:35-40.
20. Bartnicki P, Majewska E, Kowalczyk M, Baj Z, Banach M, Rysz J. Impact of anemia treatment with methoxy polyethylene glycol-epoetin beta on polymorphonuclear cells apoptosis in predialysis patients with chronic kidney disease. *Pharmacol Rep*. 2015;67:842-845. doi: 10.1016/j.pharep.2015.01.014
21. Jaber BL, Perianayagam MC, Balakrishnan VS, King AJ, Pereira BJ. Mechanisms of neutrophil apoptosis in uremia and relevance of the Fas (APO-1, CD95)/Fas ligand system. *J Leukoc Biol*. 2001;69:1006-1012.
22. Sela S, Shurtz-Swirski R, Cohen-Mazor M, Mazor R, Chezar J, Shapiro G, Hassan K, Shkolnik G, Geron R, Kristal B. Primed peripheral polymorphonuclear leukocyte: a

culprit underlying chronic low-grade inflammation and systemic oxidative stress in chronic kidney disease. *J Am Soc Nephrol*. 2005;16:2431-2438. doi: 10.1681/asn.2004110929
23. Zahran N, Sayed A, William I, Mahmoud O, Sabry O, Rafaat M. Neutrophil apoptosis: impact of granulocyte macrophage colony stimulating factor on cell survival and viability in chronic kidney disease and hemodialysis patients. *Arch Med Sci*. 2013;9:984-989. doi: 10.5114/aoms.2013.39789
24. Nahar N, Shah H, Siu J, Colvin R, Bhaskaran M, Ranjan R, Wagner JD, Singhal PC. Dialysis membrane-induced neutrophil apoptosis is mediated through free radicals. *Clin Nephrol*. 2001;56:52-59.
25. Perianayagam MC, Balakrishnan VS, Guo D, Pereira BJ, Jaber BL. Quantification of Bax and Bcl2 in polymorphonuclear leukocytes from haemodialysis patients: relation to hydrogen peroxide. *Eur J Clin Invest*. 2003;33:905-911. doi: 10.1046/j.1365-2362.2003.01225.x
26. Cohen G, Raupachova J, Wimmer T, Deicher R, Hörl WH. The uraemic retention solute para-hydroxy-hippuric acid attenuates apoptosis of polymorphonuclear leukocytes from healthy subjects but not from haemodialysis patients. *Nephrol Dial Transplant*. 2008;23:2512-2519. doi: 10.1093/ndt/gfn098
27. Guo D, Jaber BL, Lee S, Perianayagam MC, King AJ, Pereira BJ, Balakrishnan VS. Impact of iron dextran on polymorphonuclear cell function among hemodialysis patients. *Clin Nephrol*. 2002;58:134-142. doi: 10.5414/cnp58134
28. Caimi G, Montana M, Carollo C, Porretto F, Vaccaro F, Canino B, Lo Presti R. Polymorphonuclear leukocyte integrins in chronic renal failure. *Clin Hemorheol Microcirc*. 2005;32:43-49.
29. Cohen-Mazor M, Sela S, Mazor R, Ilan N, Vlodavsky I, Rops AL, van der Vlag J, Cohen HI, Kristal B. Are primed polymorphonuclear leukocytes contributors to the high heparanase levels in hemodialysis patients? *Am J Physiol Heart Circ Physiol*. 2008;294:H651-658. doi: 10.1152/ajpheart.00952.2007
30. Kim JK, Hong CW, Park MJ, Song YR, Kim HJ, Kim SG. Increased Neutrophil Extracellular Trap Formation in Uremia Is Associated with Chronic Inflammation and Prevalent Coronary Artery Disease. *J Immunol Res*. 2017;2017:8415179. doi: 10.1155/2017/8415179
31. Olsson J, Jacobson TA, Paulsson JM, Dadfar E, Moshfegh A, Jacobson SH, Lundahl J. Expression of neutrophil SOD2 is reduced after lipopolysaccharide stimulation: a potential cause of neutrophil dysfunction in chronic kidney disease. *Nephrol Dial Transplant*. 2011;26:2195-2201. doi: 10.1093/ndt/gfq673
32. Dou L, Brunet P, Dignat-George F, Sampol J, Berland Y. Effect of uremia and hemodialysis on soluble L-selectin and leukocyte surface CD11b and L-selectin. *Am J Kidney Dis*. 1998;31:67-73. doi: 10.1053/ajkd.1998.v31.pm9428454
33. Yoon JW, Pahl MV, Vaziri ND. Spontaneous leukocyte activation and oxygen-free radical generation in end-stage renal disease. *Kidney Int*. 2007;71:167-172. doi: 10.1038/sj.ki.5002019
34. Wallquist C, Mansouri L, Norrback M, Hylander B, Jacobson SH, Larsson TE, Lundahl J. Associations of Fibroblast Growth Factor 23 with Markers of Inflammation and Leukocyte Transmigration in Chronic Kidney Disease. *Nephron*. 2018;138:287-295. doi: 10.1159/000485472
35. Hassoba H, Mahmoud M, El-Gohary A. Alterations in neutrophil surface expression of L-selectin (CD62L) and soluble L-selectin (sL-selectin) in hemodialysis patients: relation to HCV. *Egypt J Immunol*. 2007;14:101-110.
36. Eldewi DM, Alhabibi AM, El Sayek HME, Ahmed S, Mahmoud K, El Sadek SM, Gouda RM, Hassan MA, Ibrahim AH, Abd El Haliem NF. Expression levels of complement regulatory proteins (CD35, CD55 and CD59) on peripheral blood cells of patients with chronic kidney disease. *Int J Gen Med*. 2019;12:343-351. doi: 10.2147/ijgm.S216989
37. Aljadi Z, Mansouri L, Nopp A, Paulsson JM, Winqvist O, Russom A, Ståhl M, Hylander B, Jacobson SH, Lundahl J. Activation of basophils is a new and sensitive marker of

biocompatibility in hemodialysis. *Artif Organs*. 2014;38:945-953. doi: 10.1111/aor.12297
38. Pereira R, Costa E, Gonçalves M, Miranda V, do Sameiro Faria M, Quintanilha A, Belo L, Lima M, Santos-Silva A. Neutrophil and monocyte activation in chronic kidney disease patients under hemodialysis and its relationship with resistance to recombinant human erythropoietin and to the hemodialysis procedure. *Hemodial Int*. 2010;14:295-301. doi: 10.1111/j.1542-4758.2010.00450.x
39. Baj Z, Zbrog Z, Szuflet A, Manka S, Bartnicki P, Majewska E. Basic inflammatory indices and chosen neutrophil receptors expression in chronic haemodialysed patients. *Central Eur J Immunol*. 2018;43:168-173. doi: 10.5114/ceji.2018.77387
40. Olsson J, Jacobson TAS, Paulsson JM, Dadfar E, Moshfegh A, Jacobson SH, Lundahl J. Expression of neutrophil SOD2 is reduced after lipopolysaccharide stimulation: a potential cause of neutrophil dysfunction in chronic kidney disease. *Nephrol Dial Transplant*. 2011;26:2195-2201. doi: 10.1093/ndt/gfq673
41. Costa E, Rocha S, Rocha-Pereira P, Nascimento H, Castro E, Miranda V, Faria Mdo S, Loureiro A, Quintanilha A, Belo L, et al. Neutrophil activation and resistance to recombinant human erythropoietin therapy in hemodialysis patients. *Am J Nephrol*. 2008;28:935-940. doi: 10.1159/000142147
42. Khatib-Massalha E, Michelis R, Trabelcy B, Gerchman Y, Kristal B, Ariel A, Sela S. Free circulating active elastase contributes to chronic inflammation in patients on hemodialysis. *Am J Physiol Renal Physiol*. 2018;314:F203-f209. doi: 10.1152/ajprenal.00070.2017
43. Otaki Y, Nakanishi T, Hasuike Y, Moriguchi R, Nanami M, Hama Y, Izumi M, Takamitsu Y. Defective regulation of iron transporters leading to iron excess in the polymorphonuclear leukocytes of patients on maintenance hemodialysis. *Am J Kidney Dis*. 2004;43:1030-1039. doi: 10.1053/j.ajkd.2004.02.016
44. Capeillere-Blandin C, Gausson V, Nguyen AT, Descamps-Latscha B, Drueke T, Witko-Sarsat V. Respective role of uraemic toxins and myeloperoxidase in the uraemic state. *Nephrol Dial Transplant*. 2006;21:1555-1563. doi: 10.1093/ndt/gfl007
45. Tsai MS, Shaw HM, Li YJ, Lin MT, Lee WT, Chan KS. Myeloperoxidase in chronic kidney disease: Role of visceral fat. *Nephrology*. 2014;19:136-142. doi: 10.1111/nep.12187
46. Deicher R, Exner M, Cohen G, Haag-Weber M, Hörl WH. Neutrophil beta(2)-microglobulin and lactoferrin content in renal failure patients. *Am J Kidney Dis*. 2000;35:1117-1126. doi: 10.1016/s0272-6386(00)70049-7
47. Jeong JC, Kim JE, Gu JY, Yoo HJ, Ryu JW, Kim DK, Joo KW, Kim HK. Significance of the DNA-Histone Complex Level as a Predictor of Major Adverse Cardiovascular Events in Hemodialysis Patients: The Effect of Uremic Toxin on DNA-Histone Complex Formation. *Blood Purif*. 2016;41:64-71. doi: 10.1159/000440974
48. Kim JK, Park MJ, Lee HW, Lee HS, Choi SR, Song YR, Kim HJ, Park HC, Kim SG. The relationship between autophagy, increased neutrophil extracellular traps formation and endothelial dysfunction in chronic kidney disease. *Clin Immunol*. 2018;197:189-197. doi: 10.1016/j.clim.2018.10.003
49. Vega-Roman C, Leal-Cortes C, Portilla-de Buen E, Gomez-Navarro B, Melo Z, Franco-Acevedo A, Medina-Perez M, Jalomo-Martinez B, Martinez-Martinez P, Evangelista-Carrillo LA, et al. Impact of transplantation on neutrophil extracellular trap formation in patients with end-stage renal disease: A single-center, prospective cohort study. *Medicine (Baltimore)*. 2021;100:e26595. doi: 10.1097/md.0000000000026595
50. Dahlgren C, Karlsson A, Bylund J. Intracellular Neutrophil Oxidants: From Laboratory Curiosity to Clinical Reality. *J Immunol*. 2019;202:3127-3134. doi: 10.4049/jimmunol.1900235
51. Ward RA, Ouseph R, McLeish KR. Effects of high-flux hemodialysis on oxidant stress. *Kidney Int*. 2003;63:353-359. doi: 10.1046/j.1523-1755.2003.00741.x
52. Paul JL, Roch-Arveiller M, Man NK, Luong N, Moatti N, Raichvarg D. Influence of uremia on polymorphonuclear leukocytes oxidative metabolism in end-stage renal disease and dialyzed patients. *Nephron*. 1991;57:428-432. doi: 10.1159/000186308

53. Kiersztejn M, Smogorzewski M, Thanakitcharu P, Fadda GZ, Massry SG. Decreased O2 consumption by PMNL from humans and rats with CRF: role of secondary hyperparathyroidism. *Kidney Int*. 1992;42:602-609. doi: 10.1038/ki.1992.324
54. Rao M, Guo D, Jaber BL, Sundaram S, Cendoroglo M, King AJ, Pereira BJ, Balakrishnan VS. Dialyzer membrane type and reuse practice influence polymorphonuclear leukocyte function in hemodialysis patients. *Kidney Int*. 2004;65:682-691. doi: 10.1111/j.1523-1755.2004.00429.x
55. Cohen G, Raupachova J, Ilic D, Werzowa J, Hörl WH. Effect of leptin on polymorphonuclear leucocyte functions in healthy subjects and haemodialysis patients. *Nephrol Dial Transplant*. 2011;26:2271-2281. doi: 10.1093/ndt/gfq731
56. Cohen-Hagai K, Nacasch N, Sternschuss A, Ohana M, Wolach B, Benchetrit S, Gavrieli R, Zitman-Gal T. Malnutrition and inflammation in hemodialysis patients: Comparative evaluation of neutrophil reactive oxygen formation. *Nutrition*. 2020;78:110793. doi: 10.1016/j.nut.2020.110793
57. Dobos GJ, Traynor-Kaplan AE, Ward D, Schollmeyer PJ. Neutrophil dysfunction in end-stage renal failure: reduced response to priming by C5a. *Clin Investig*. 1994;72:353-357. doi: 10.1007/bf00252827
58. Ward R, McLeish KR. Soluble TNF alpha receptors are increased in chronic renal insufficiency and hemodialysis and inhibit neutrophil priming by TNF alpha. *Artif Organs*. 1996;20:390-395. doi: 10.1111/j.1525-1594.1996.tb04521.x
59. Haynes AP, Daniels I, Porter C, Fletcher J, Morgan AG. Abnormal cytoplasmic pH regulation during activation in uremic neutrophils. *Kidney Int*. 1992;42:690-699. doi: 10.1038/ki.1992.336
60. Kristal B, Shurtz-Swirski R, Shasha SM, Manaster J, Shapiro G, Furmanov M, Hassan K, Weissman I, Sela S. Interaction between erythropoietin and peripheral polymorphonuclear leukocytes in hemodialysis patients. *Nephron*. 1999;81:406-413. doi: 10.1159/000045324
61. Ward RA, McLeish KR. Soluble TNF alpha receptors are increased in chronic renal insufficiency and hemodialysis and inhibit neutrophil priming by TNF alpha. *Artif Organs*. 1996;20:390-395. doi: 10.1111/j.1525-1594.1996.tb04521.x

Supplementary tables

Suppl. Table 1. Search strategy, in- and exclusion criteria for the identification of studies investigating neutrophil function in CKD patients. This study was performed according to the Preferred Reporting Items for Systematic reviews and Meta-Analyses (PRISMA) guidelines (not registered).

**SYSTEMATIC REVIEW: Search strategy**
PubMed:<br>((("neutrophils"[MeSH Terms]) OR ("granulocytes"[MeSH Terms])) AND (renal insufficiency, chronic[MeSH Terms])) AND (("1990"[Date - Publication] : "3000"[Date - Publication]))
Web of Science:<br>((AB=(neutrophils)) OR (AB=(granulocytes)))<br>AND<br>((TS=("chronic renal insufficiency")) OR (TS=(CKD)) OR (TS=("chronic kidney disease")))<br>AND<br>PY=(1990-2023)<br>With "exact search"
**SYSTEMATIC REVIEW: In- and exclusion criteria during title and abstract screening (first stage)**
Inclusion criteria
- if neutrophil function in blood of patients with CKD was assessed in comparison to that of healthy volunteers
Exclusion criteria
- if written in another language than English<br>- if the work was non-original or only conference abstracts were available<br>- if being a meta-analysis or systemic review<br>- if being an ongoing clinical trial, study protocol or case report<br>- if exclusively dealing with animal studies<br>- if only specific underlying pathologies of CKD were investigated (e.g. lupus nephritis) or no CKD focus was included (no CKD or too specific CKD pathology)<br>- if neutrophils were not investigated
**SYSTEMATIC REVIEW: Additional exclusion criteria during full text assessment (second stage)**
- if the full text could not be accessed<br>- if using patient material other than blood<br>- if only including transplanted patients<br>- if only measuring neutrophil-to-lymphocyte-ratio or neutrophil numbers<br>- if lacking a healthy control group<br>-if they used non-adult control group vs. adult experimental group<br>- if only specific underlying pathologies of CKD were investigated (e.g. lupus nephritis, cancer associated) or no CKD focus was included (no CKD or too specific CKD pathology)
**META-ANALYSIS of ROS production: Additional exclusion criteria (third stage)**
Inclusion criteria
- if studies analyzed neutrophil ROS production in CKD patients on dialysis compared to healthy controls

Exclusion criteria
- if studies used an indirect measurement of ROS like oxygen consumption via Clarke electrode or measurement of CO_2 production via radiolabeled glucose (incomparable assay of ROS analysis used)<br>- resulting data only given as median<br>- patients reported to be newly started on dialysis<br>- studies that did not fulfill the criterium of certainty in the body of evidence for ROS analysis<br>- number of included controls/patients unclear (missing experimental details)<br>- controls and patients not clearly defined<br>- study population not clearly defined

Suppl. Table 2. Summary of overall outcome of neutrophil activity parameters in patients with chronic kidney disease compared to healthy controls. Studies are counted towards a certain category (decreased/unaltered/increased) if one of their included datasets showed the respective outcome and may therefore be included in different categories; the sum of all categories (decreased/unaltered/increased) can therefore exceed the total number of studies per readout. *fMLP, N-formyl-methionyl-leucyl-phenylalanine; LPS, lipopolysaccharides; NETs, neutrophil extracellular traps; PAMPS, Pathogen-associated molecular patterns; PMA, phorbol-12-myristate-13-acetate; ROS, reactive oxygen species.*

**Parameters**	**Nr. of studies**	**Decreased**		**Unaltered**		**Increased**	
		**Nr.**	**Ref.**	**Nr**	**Ref.**	**Nr.**	**Ref.**
Phagocytosis	19	13	[1-13]	13	[3,4,6,10-19]	0	
Apoptosis	9	0	-	5	[4,21,25-27]	6	[4,20-24]
Surface activation markers – Basal	14	3	*(Including studies with increased CD62L, reflecting decreased neutrophil activation)* [35,36,38]	8	[8,28,30,32,34,37-39]	10	*(Including studies with decreased CD62L, reflecting increased neutrophil activation)* [8,23,28-35]
Surface activation markers – Stimulated neutrophils	4	0	-	3	[28,34,37]	3	[28,31,34]
Degranulation	8	1	[43]	4	[41,44,46,47]	8	[8,41-47]
NET formation-basal	4	0		2	[8,49]	2	[30,48]
NET formation-stimulated neutrophils	4	1	[8]	2	[30,49]	1	[48]
ROS production-Basal	13	1	[53]	7	[3,4,18,43,44,51,52]	7	[3,15,25,27,30,33,44]
ROS production-Stimulated by:	20	9		7		10	
PAMPs (E.Coli/LPS, S. aureus, C. albicans, Zymosan)	11	4	[8,22,55,56]	4	[3,4,10,14]	5	[3,4,27,51,52]
PMA	12	5	[8,15,54-56]	3	[4,18,27]	4	[22,29,52,60]
fMLP	7	2	[61](fMLP+TNFα) [53]	2	[4,54]	4	[4,18,27,59]
Complement	1	1	[57](C5a +FLPEP)	0	-	0	-

Suppl. Table 3. Summary of studies on neutrophil phagocytosis capacity in patients with CKD compared to healthy controls. Studies comparing non-dialysis and dialysis CKD patients are highlighted in grey. *CKD, chronic kidney disease; SMC, synthetically modified cellulose; CL-E, vitamin E-modified membrane; PS, polysulfone membrane; HD, hemodialysis; PD, peritoneal dialysis.*

Author	Year	PMID	CKD Stage	Dialysis type (for CKD5 patients)	Phagocytotic activity: target	Method used	Author conclusions	Increased/ decreased/ unaltered *phagocytosis* (CKD patients vs. healthy controls)
Alexiewicz et al.	1991	1951470	5	HD	*E.Coli*	Analysis of uptake of labeled E.Coli by spectrophotometry	Significantly impaired	Decreased
Alexiewicz et al.	1995	7872322	5	HD	*E.coli*	Analyis of uptake of oil droplets coated with E. coli LPS	Significantly impaired	Decreased
Anding et al.	2003	13679482	5	HD	*C.albicans*	Analysis of uptake of labeled C.albicans by flow cytometry	Slightly lower, but not statistically significant	Unaltered
Banche et al.	2006	16935897	5	HD	*K. pneumoniae*	Analysis of uptake of labeled K. pneumoniae by spectrophotometry	Compared to healthy controls, unaltered in HD when using CL-E or PS membrane but decreased when using SMC dialysis membrane	Decreased in HD when using SMC dialysis membrane<br>Unaltered in HD when using a CL-E and PS membrane
Cuffini et al.	2001	11382696	5	HD	*K.pneumoniae*	Analysis of uptake of labeled K.pneumoniae by spectrophotometry	Lower phagocytosis after 30 and 90 min bacterial stimulation	Decreased
Lemesch et al.	2016	27698480	3 to 5	HD and PD	*E.coli*	Analysis of uptake of labeled E.coli by flow cytometry	Significantly lower in patients undergoing HD, but not in PD. No differences in non-dialysis CKD	Unaltered in non-dialysis CKD and PD<br>Decreased in HD
Mahajan et al.	2005	16060119	2 to 5	HD	*IgG coated sheep erythrocytes*	Analysis of uptake of IgG-coated sheep erythrocytes by spectrophotometry	Compared to controls, significantly reduced in patients undergoing HD but not in non-dialysis CKD. Significantly reduced before compared to after HD session.	Unaltered in non-dialysis CKD<br>Decreased in HD
Muniz-Junqueira et al.	2005	16005905	Non-dialysis not specified and 5	HD and PD	*S.cerevisiae*	Analysis of uptake of labeled S. cerevisiae by spectrophotometry	Significantly lower in patients undergoing HD, but not in PD. No differences in non-dialysis CKD	Unaltered in non-dialysis CKD and PD<br>Decreased in HD
Pappas et al.	2019	31605588	5	HD	*E.Coli*	Analysis of uptake of labeled E.Coli by flow cytometry	Significantly impaired	Decreased
Patruta et al.	1998	9555668	5	HD	*E.coli*	Killing capacity by fluorescence microscopy	Lower, but not statistically significant	Unaltered
Peng et al.	2014	25339482	Not specified	Not specified	*Not specified yeasts*	Killing capacity by microscopy	Significantly impaired	Decreased
Porter et al.	1995	8569942	Non-dialysis CKD 5	PD	*S. epidermidis*	Analysis of uptake of labeled S. epidermidis by spectrophotometry	Decreased in non-dialysis CKD5 when compared to control. No difference between PD patients and controls.	Decreased in non-dialysis CKD5<br>Unaltered in PD
Porter et al.	1997	9434073	5	PD	*S. epidermidis*	Killing capacity by fluorescence microscopy	Decreased in non-dialysis CKD5 when compared to control as well as PD patients. No difference between PD patients and controls.	Decreased in non-dialysis CKD5<br>Unaltered in PD
Sardenberg et al.	2006	16155068	Non-dialysis not specified and 5	HD and PD	*S.aureus*	Analysis of uptake of labeled S.aureus by flow cytometry	Significantly lower in patients undergoing HD, but not in PD. No differences in non-dialysis CKD	Unaltered in non-dialysis CKD and PD<br>Decreased in HD
Scalas et al.	2012	24712758	5	HD	*C.albicans*	Analysis of uptake of labeled C.albicans by flow cytometry	No significant difference	Unaltered
Schauer et al.	1991	1775265	5	HD	*S.cerevisiae*	Analysis of uptake of labeled S. cerevisiae by spectrophotometry	No significant difference	Unaltered
Talal et al.	2022	36284314	5	HD	*E.Coli*<br>*S.aureus*	Analysis of uptake of labeled E.Coli and S.aureus by spectrophotometry	Significantly impaired	Decreased
Tullio et al.	2003	12868562	5	HD	*C.albicans*	Analysis of uptake of labeled C.albicans by flow cytometry	No significant difference	Unaltered
Ward et al.	1995	7780059	Not specified	Not specified	*S. aureus*	Analysis of uptake of labeled S.aureus by flow cytometry	No significant difference	Unaltered

Suppl. Table 4. Summary of studies on neutrophil apoptosis in patients with CKD compared to healthy controls. Studies comparing non-dialysis and dialysis CKD patients are highlighted in grey. CKD, chronic kidney disease. *HC, healthy control; HD, hemodialysis; PD, peritoneal dialysis.*

Author	PMID	Year	CKD Stage	Dialysis type (for CKD5 patients)	Method used	Author conclusions	Increased/ decreased/ unaltered *apoptosis* (CKD patients vs. healthy controls)
Bartnicki et al.	26398374	2015	4 and 5	Non-dialysis	Flow cytometry: Annexin V/Propidium Iodide (PI)<br>Fas and FasL expression	Annexin+ and PI+: increased<br>Fas and FasL: increased	Increased
Cohen et al.	18319358	2008	5	HD	Flow cytometry: Spontaneous apoptosis asssay (viability x Propidium Iodide)	Spontaneous apoptosis: unaltered	Unaltered
Guo et al.	12227686	2002	5	HD	Flow cytometry: Annexin V/Propidium Iodide (PI)	Annexin+ and PI+: unaltered	Unaltered
Jaber et al.	11404388	2001	Non-dialysis not specified and 5	HD and PD	Flow cytometry: Annexin V<br>Fas-FasL expression	Annexin V: increased in non-dialysis CKD, unaltered in HD and PD (compared to control)<br>Fas-FasL: increased in non-dialysis CKD, unaltered in HD and PD	Increased in non-dialysis CKD<br>Unaltered in HD and PD
Nahar et al.	11499659	2001	5	HD	Propidium Iodide (PI) fluorescence analysis	PI: increased	Increased
Perianayagam et al.	14511363	2003	5	HD	Flow cytometry: Propidium Iodide (PI), Bax (pro-apoptotic), Bcl2 (anti-apoptotic)	PI: unaltered<br>Bax-Bcl2: more frequently detected in HD	Unaltered
Sardenberg et al.	16155068	2006	Non-dialysis not specified and 5	HD and PD	Flow cytometry: Annexin V	Annexin: increased in non-dialysis CKD but unaltered in HD and PD (compared to HC)	Increased in non-dialysis CKD<br>Unaltered in HD and PD
Sela et al.	15987755	2005	2 to 5	HD and PD	Flow cytometry: Annexin V	Annexin: increased	Increased in both non-dialysis, HD and PD patients
Zahran et al.	24482640	2013	Non-dialysis not specified and 5	HD	Flow cytometry: AnnexinV<br>Morphological apoptosis: chromatin condensation, fragmented nuclei, cell shrinkage and the presence of cytoplasmic vacuolization	Annexin: increased<br>Morphological apoptosis: Increased	Increased in both non-dialysis and dialysis patients

Suppl. Table 5. Summary of studies on neutrophil surface markers in patients with CKD compared to healthy controls. A) Surface markers studied on neutrophils in the identified manuscripts. **B)** Neutrophils were studied in basal conditions or upon stimulation, as indicated. Studies comparing non-dialysis and dialysis CKD patients are highlighted in grey. *CKD, chronic kidney disease; fMLP, N-formyl-methionyl-leucyl-phenylalanine; HD, hemodialysis; IL-8, interleukin 8; LPS, lipopolysaccharides; PD, peritoneal dialysis; PMA, phorbol myristate acetate.*

A)

Surface marker	Expected upon neutrophil activation
CD11b (integrin chain)	Upregulated
CD11a (integrin chain)	Upregulated
CD18 (integrin chain)	Upregulated
CD88 (C5aR1)	Upregulated
CD66b	Upregulated
CD35 (Complement receptor type 1)	Upregulated
CD62L (L-selectin)	Downregulated (shedded)

Surface receptor	Information
CD15	Neutrophil marker
CXCR1	Receptor for CXCL8 (IL8)
CXCR2	Receptor for CXCL8 (IL8)
CD14	Receptor for endotoxin

B)

**CD62L: is shed upon neutrophil activation*

Author	Year	PMID	CKD Stage	Dialysis type (for CKD5 patients)	Method used	Surface activation markers	Stimuli	Author conclusions	Basal Increased/decreased/unaltered *neutrophil activation* (comparing patients to healthy controls)	Stimulated Increased/decreased/unaltered *neutrophil activation* (comparing patients to healthy controls)
Aljadi et al.	2014	24712758	5	HD	Flow cytometry	CD11b, CD88	IL-8	Basal: No significant differences<br>Stimulated with IL-8: No significant differences (Control x HD), but increased CD11b compared to unstimulated	Basal (CD11b and CD88): unaltered	Stimulated (CD11b) : unaltered
Baj et al.	2018	30135629	5	HD	Flow cytometry	CD14, TLR4, TLR9	Only basal	Basal: No significant differences for TLR4 and 9	Basal (TLR4,9): unaltered	-
Caimi et al.	2005	15665425	Not specified	Not specified	Flow cytometry	CD11a, CD11b, CD18	PMA<br>fMLP	Basal: increased CD11b and CD18. Unaltered CD11a. Stimulated with PMA and fMLP: increased CD11b and CD18. No significant differences for CD11a	Basal (CD11b and CD18): increased<br>Basal (CD11a): unaltered	Stimulated (CD11b and CD18): increased<br>Stimulated (CD11a): unaltered
Cohen-Mazor et al.	2008	18319358	5	HD	Flow cytometry	CD11b	Only basal	Basal: increased	Basal (CD11b): increased	-
Dou et al.	1998	9428454	Non-dialysis not specified and 5	HD	Flow cytometry	CD62L, CD11b	Only basal	Basal non-dialysis CKD: increased CD11b, decreased CD62L<br>Basal HD (before dialysis session): unaltered	Basal (CD11b): increased in non-dialysis CKD but unaltered in HD<br>Basal (CD62L): decreased CD62L reflecting increased activation* in non-dialysis CKD but unaltered in HD	-
Eldewi et al.	2019	31571973	Non-dialysis 3-5	-	Flow cytometry	CD35	Only basal	Basal: decreased	Basal (CD35): decreased	-
Hassoba et al.	2007	20306662	5	HD	Flow cytometry	CD62L	Only basal	Basal: increased compared to controls, but decreased levels after dialysis session	Basal (CD62L): increased CD62L, reflecting reduced activation*<br>After HD session: decreased CD62L, reflecting increased activation*	-
Kim et al.	2017	28523279	5	HD	Flow cytometry	CD35 and CD66b	Only basal	Basal: No significant differences (CD63 and CD66b) and increased (CD35)	Basal (CD63, CD66b): unaltered<br>Basal (CD35): increased	-
Olsson et al.	2011	21045076	Non-dialysis 3-5	-	Flow cytometry	CD11b	LPS	Basal: increased<br>Stimulated with LPS: significant increase in both patients with CKD and healthy controls	Basal (CD11b): increased	Stimulated (CD11b): increased
Pereira et al.	2010	20649681	5	HD	Flow cytometry	CD11b and CXCR1	Only basal	Basal: decreased CXCR1 and no significant differences for CD11b	Basal (CXCR1): decreased<br>Basal (CD11b): unaltered	-
Talal et al.	2022	36284314	5	HD	Flow cytometry	CD18, CD11b, CD66b, CD15	Only basal	Basal: increased CD18, CD66b and CD15. No significant differences for CD11b	Basal (CD18, CD66b, CD15): increased<br>Basal (CD11b): unaltered	-
Wallquist et al.	2018	29301137	Non-dialysis 2–5	-	Flow cytometry	CD11b and CD62L	fMLP	Basal: increased CD11b and no significant differences for CD62L	Basal (CD11b): increased<br>Basal (CD62L*): unaltered	Stimulated (CD11b): increased<br>Stimulated (CD62L): unaltered
Yoon et al.	2007	17136029	5	HD	Flow cytometry	CD18, CD11b	Only basal	Basal: increased	Basal (CD11b): increased<br>Basal (CD18): increased	-
Zahran et al.	2013	24482640	Non-dialysis not specified and 5	HD	Flow cytometry	CD18	Only basal	Basal non-dialysis CKD and HD: increased	Basal (CD18): increased	-

Suppl. Table 6. Summary of studies on neutrophil degranulation in patients with CKD compared to healthy controls. Studies comparing non-dialysis and dialysis CKD patients are highlighted in grey. *CKD, chronic kidney disease; HD, hemodialysis; MMP9, matrix metalloproteinase-9; MPO, myeloperoxidase; NE, neutrophil elastase; fMLP, N-formyl-methionyl-leucyl-phenylalanine.*

Author	Year	PMID	CKD Stage	Dialysis type (for CKD5 patients)	Method used	Author conclusions	Based on PLASMA/SERUM markers: Increased/ decreased/ unaltered *degranulation* (CKD patients vs. healthy controls)	Based on ISOLATED NEUTROPHIL readouts: Increased/ decreased/ unaltered *degranulation* (CKD patients vs. healthy controls)
Capeillère-Blandin et al.	2006	16476719	2 to 5	HD	Plasma levels of MPO (by ELISA)	No significant differences in non-dialysis CKD, but significantly increased in HD patients	Unaltered in non-dialysis CKD<br>Increased in HD	-
Costa et al.	2008	18587235	5	HD	Plasma levels of neutrophil elastase and lactoferrin by ELISA	Significantly higher elastase and unaltered lactoferrin in plasma	Elastase: increased<br>Lactoferrin: unaltered	-
Deicher et al.	2000	10845826	5	HD	Plasma levels of lactoferrin and its release upon PMA stimulation of isolated neutrophils by ELISA	Significantly higher levels in plasma, but no significant differences in isolated neutrophils	Increased	Unaltered release (upon PMA stimulation)
Jeong et al.	2016	29130984	5	HD	Plasma levels of histone-DNA, cell-free DNA and neutrophil elastase (NE) by ELISA	Significantly higher levels of histone-DNA, but no significant differences of cell-free DNA and NE	Histone-DNA: Increased<br>Cell-free DNA and NE: Unaltered	-
Khatib-Massalha et al.	2018	29046295	5	HD	Neutrophil elastase: Serum levels (by ELISA); activity, intracellular level (by western blotting) and surface level (by flow cytometry) in isolated neutrophils	Significantly higher serum levels, surface markers and decreased intracellular level, and activity of neutrophil elastase (more degranulation)	Increased	Reduced basal content (due to increased prior degranulation) (basal)
Otaki et al.	2004	15168383	5	HD	Release of MPO, NE and lactoferrin upon fMLP stimulation of isolated neutrophils (by ELISA)	Significantly higher MPO and NE release and reduced lactoferrin release after stimulation with fMLP	-	MPO and NE: Increased release (upon fMLP stimulation)<br>Lactoferrin: decreased (upon fMLP stimulation)
Talal et al.	2022	36284314	5	HD	Protein levels of MPO, MMP9 and neutrophil elastase in isolated neutrophils	Significantly lower (more degranulation)	-	Reduced basal content (due to increased prior degranulation) (basal)
Tsai et al.	2013	24330098	Not specified	Not specified	Plasma levels of MPO (by ELISA)	Significantly higher	Increased	-

Suppl. Table 7. Summary of studies on neutrophil NET formation in patients with CKD compared to healthy controls. Neutrophils were studied in basal conditions or upon stimulation, as indicated. *CKD, chronic kidney disease; H3Cit, citrullinated histone H3; HD, hemodialysis; MPO, myeloperoxidase; NET, neutrophil extracellular traps; PD, peritoneal dialysis; PMA, phorbol myristate acetate.*

Author	Year	PMID	CKD Stage	Dialysis type (for CKD5 patients)	Method used	Stimuli	Author conclusions	Basal Increased/decreased/unaltered *NETosis* (comparing patients to healthy controls)	Stimulated Increased/decreased/unaltered *NETosis* (comparing patients to healthy controls)
Kim et al.	2017	1405338	5	HD	NET formation assay using Sytox green in isolated neutrophils	Basal<br>PMA	Basal: significantly increased<br>Stimulated with PMA: no significant differences	Increased	Unaltered (but with lower fold-increase of stimulated vs. unstimulated)
Kim et al.	2018	30296592	5	HD	Quantification of NET-releasing neutrophils via immunofluorescence of MPO and H3Cit from isolated neutrophils	Basal<br>PMA	Significantly increased NETosis via MPO staining but not via H3Cit (low signal)<br>Stimulated with PMA: Significantly increased NETosis via both MPO and H3Cit signal	Increased	Increased
Talal et al.	2022	36284314	5	HD	NET formation assay using Sytox green in isolated neutrophils and supernatant after PMA stimulation	Basal<br>Calcium ionophore<br>PMA	Basal: no significant differences<br>Stimulated with calcium ionophore: significantly decreased DNA release compared to stimulated healthy cells, but increased compared to CKD untimulated cells.<br>Stimulated with PMA: significantly decreased DNA release compared to stimulated healthy cells, but no differences compared to CKD untimulated cells	Unaltered	Decreased
Vega-Roman et al.	2021	2123637	5	Mixed: mainly HD/PD with few non-dialysis	NET formation assay using Sytox green in isolated neutrophils	Basal<br>PMA	Basal: no significant differences<br>Stimulated with PMA: no significant differences between healthy or CKD, only when comparing unstimulated with PMA-treated cells	Unaltered	Unaltered

Suppl. Table 8. Summary of studies on neutrophil ROS production in patients with CKD compared to healthy controls. Neutrophils were studied in basal conditions or upon stimulation, as indicated. Studies comparing non-dialysis and dialysis CKD patients are highlighted in grey. *CKD, chronic kidney disease; fMLP, N-formyl-methionyl-leucyl-phenylalanine; HD, hemodialysis; PMNL, polymorphonuclear neutrophil leukocytes; PD, peritoneal dialysis; PMA, phorbol myristate acetate; TNFα, tumor necrosis factor alpha.*

Study	PMID	Patient cohort		Neutrophils in full blood/isolated neutrophils	ROS Assay	Stimulus	Overall study conclusion on ROS response			
		Non-dialysis	Dialysis				Non-dialysis patients vs. healthy controls	Dialysis patients vs. healthy controls	Dialysis patients vs. non-dialysis	HD vs. PD
Anding et al., 2003	13679482	-	HD	Full blood analysis	Dihydrorhodamine	C.albicans	-	Unaltered (stimulated)	-	-
Capeillere-Blandin et al. 2006	16476719	CKD 2-4	HD	Full blood analysis	Lucigenin enhanced chemiluminescence	No stimuli	Unaltered (unstimulated)	Increased (unstimulated)	Increased (unstimulated)	-
Cohen et al., 2011	21216885	-	HD	Full blood analysis	Dihydrorhodamine	E.coli<br>PMA	-	Decreased (stimulated)	-	-
Cohen-Hagai et al., 2020	32454385	-	HD	PMNL	Dihydrorhodamine	E.coli<br>PMA	-	Decreased (stimulated)	-	-
Cohen-Mazor et al., 2008	18032524	-	HD	PMNL	Cytochrome c reduction	PMA	-	Increased (stimulated)	-	-
Dobos et al., 1994	8086769	-	HD	PMNL	Fluorescent para-hydroxyphenylacetic acid assay	C5a + FLPEP	-	Decreased (stimulated)	-	-
Guo et al., 2002	12227686	-	HD	PMNL	Dichlorofluorescein diacetate (DCFH-DA)	PMA<br>fMLP<br>S.aureus	-	Increased (unstimulated and stimulated S.aureus and fMLP)<br>Unaltered (stimulated PMA)	-	-
Haynes et al. 1992	1328756	CKD 5	PD	PMNL	Lucigenin enhanced chemiluminescence	fMLP	Increased (stimulated)	Increased (stimulated)	Unaltered (stimulated)	-
Kierszteijn et al. 1992	1405338	-	HD	PMNL	Oxygen consumption (as readout of respiratory burst)	fMLP	-	Decreased (unstimulated and stimulated)	-	-
Kim et al. 2017	28523279	-	HD	PMNL	Dichlorofluorescein diacetate (DCFH-DA)	No stimuli	-	Increased (unstimulated)	-	-
Kristal et al., 1998	10095176	-	HD	PMNL	Cytochrome c reduction	PMA	-	Increased (stimulated)	-	-
Lemesch et al. 2016	27698480	CKD 3-5	HD and PD	PMNL	Ingestion of fluorescein-isothiocyanate (FITC) labelled opsonized bacteria	E.Coli	Unaltered (unstimulated and stimulated)	Increased (in HD, unstimulated and stimulated)<br>Unaltered (in PD, unstimulated and stimulated)	Unaltered (stimulated)	Increased
Otaki et al., 2004	15168383	-	HD	PMNL	Dichlorofluorescein diacetate (DCFH-DA)	No stimuli	-	Unaltered (unstimulated)	-	-
Patruta et al., 1998	9555668	-	HD	PMNL	Cytochrome c reduction	PMA	-	Increased (unstimulated)<br>Decreased (stimulated)	-	-
Perianayagam et al., 2003	14511363	-	HD	Full blood analysis	Dichlorofluorescein diacetate (DCFH-DA)	No stimuli	-	Increased (unstimulated)	-	-
Paul et al., 1991	1646409	-	HD	PMNL	Cytochrome c reduction	PMA<br>zymosan	-	Unaltered (unstimulated)<br>Increased (stimulated)	-	-
Porter et al., 1997	9434073	Not specified non-dialysis CKD	PD	PMNL	Dichlorofluorescein diacetate (DCFH-DA)	S.epidermidis	Decreased (stimulated)	Unaltered (stimulated)	Unaltered (stimulated)	-
Rao et al., 2004	14717942	-	HD	PMNL	Cytochrome c reduction	PMA<br>fMLP	-	Decreased (stimulated PMA)<br>Unaltered (stimulated fMLP)	-	-
Sardenberg et al. 2006	16155068	Not specified non-dialysis CKD	HD and PD	Full blood analysis	Dichlorofluorescein diacetate (DCFH-DA)	S.aureus<br>fMLP<br>PMA	Unaltered (unstimulated and stimulated)	Unaltered (in HD)<br>Increased (in PD; S. aureus, fMLP)	Unaltered (in HD)<br>Increased (in PD; S. aureus, fMLP, unstimulated)	Decreased (S. aureus, fMLP, unstimulated)
Sela et al. 2005	15987755	CKD 2-5	HD and PD	PMNL	Cytochrome c reduction	PMA<br>Zymosan	Increased (stimulated PMA)<br>Decreased (stimulated Zymosan)	Increased (stimulated PMA)<br>Decreased (stimulated Zymosan)	Increased (in HD, stimulated PMA)<br>Unaltered (in PD, stimulated PMA)<br>Unaltered (stimulated Zymosan)	Unaltered
Talal et al., 2022	36284314	-	HD	Neutrophils	"ROS-ID" total ROS/ Superoxide detection kit	PMA<br>LPS	-	Decreased (stimulated)	-	-
Ward et al. 1996	8725616	Not specified non-dialysis azotemic patients	HD	PMNL	Cytochrome c reduction	fMLP<br>TNFα + fMLP	Unaltered (fMLP)<br>Decreased (TNFα + fMLP)	Unaltered (fMLP)<br>Decreased (TNFα + fMLP)	Unaltered (stimulated)	-
Ward et al. 1995	7780059	Not specified non-dialysis CKD	-	PMNL	Cytochrome c reduction	fMLP<br>PMA	Unaltered (unstimulated)<br>Increased (stimulated fMLP)<br>Unaltered (stimulated PMA)	-	-	-
Ward et al.2003	12472803	-	HD	Full blood analysis	H_2O_2 measurement	S.aureus	-	Increased (unstimulated and stimulated)	-	-
Yoon et al., 2007	17136029	-	HD	Full blood analysis	Dihydroethidium (DHE), Dihydrorhodamine oxidation	No stimuli	-	Increased (unstimulated)	-	-

Suppl. Table 9. Summary of assays used by studies in the meta-analysis to measure ROS production of neutrophils. Different assays measure different types of ROS in different cellular locations with varying specificity. *DCFH-DA, 2',7'-dichlorodihydrofluorescein diacetate; DHE, dihydroethidium; DHR, dihydrorhodamine; FC, flow cytometry; ROS, reactive oxygen species; SOD, superoxide dismutase.*

<table>
<tr><th>ROS probe</th><th>Cellular location</th><th>Specificity</th><th>Type of ROS</th></tr>
<tr><td>DHR oxidation (Flow cytometry)</td><td>Intracellular</td><td>Non-specific</td><td>Reactive nitrogen species (e.g. peroxynitrite), hydrogen peroxide (indirectly via peroxidases), hypochlorous acid</td></tr>
<tr><td>DHE oxidation (Flow cytometry)</td><td>Intracellular</td><td>Non-specific</td><td>Superoxide, plus non-specific oxidation</td></tr>
<tr><td>DCFH-DA oxidation (Flow cytometry)</td><td>Intracellular</td><td>Non-specific</td><td rowspan="2">Diverse ROS, including hydrogen peroxide (indirectly via peroxidases), lipid hydroperoxides, peroxynitrite and other oxidants, but minimally reactive with superoxide</td></tr>
<tr><td>DCFH-DA oxidation (Plate reader)</td><td>Total</td><td>Non-specific</td></tr>
<tr><td>Luminol-enhanced chemiluminescence</td><td>Total</td><td>Partially specific</td><td>Particularly superoxide and hydrogen peroxide (indirectly via peroxidases)</td></tr>
<tr><td>Lucigenin-enhanced chemiluminescence</td><td>Extracellular</td><td>Partially specific (but prone to artefacts, including superoxide production)</td><td>Primarily extracellular superoxide when used with an SOD-inhibitor</td></tr>
<tr><td>SOD-inhibitable cytochrome c reduction</td><td>Extracellular</td><td>Partially specific</td><td>Primarily extracellular superoxide</td></tr>
</table>

Superoxide (O_2^-), hydrogen peroxide (H_2O_2), peroxynitrite ($ONOO^-$), hypochlorous acid (HOCl)

Supplementary figures

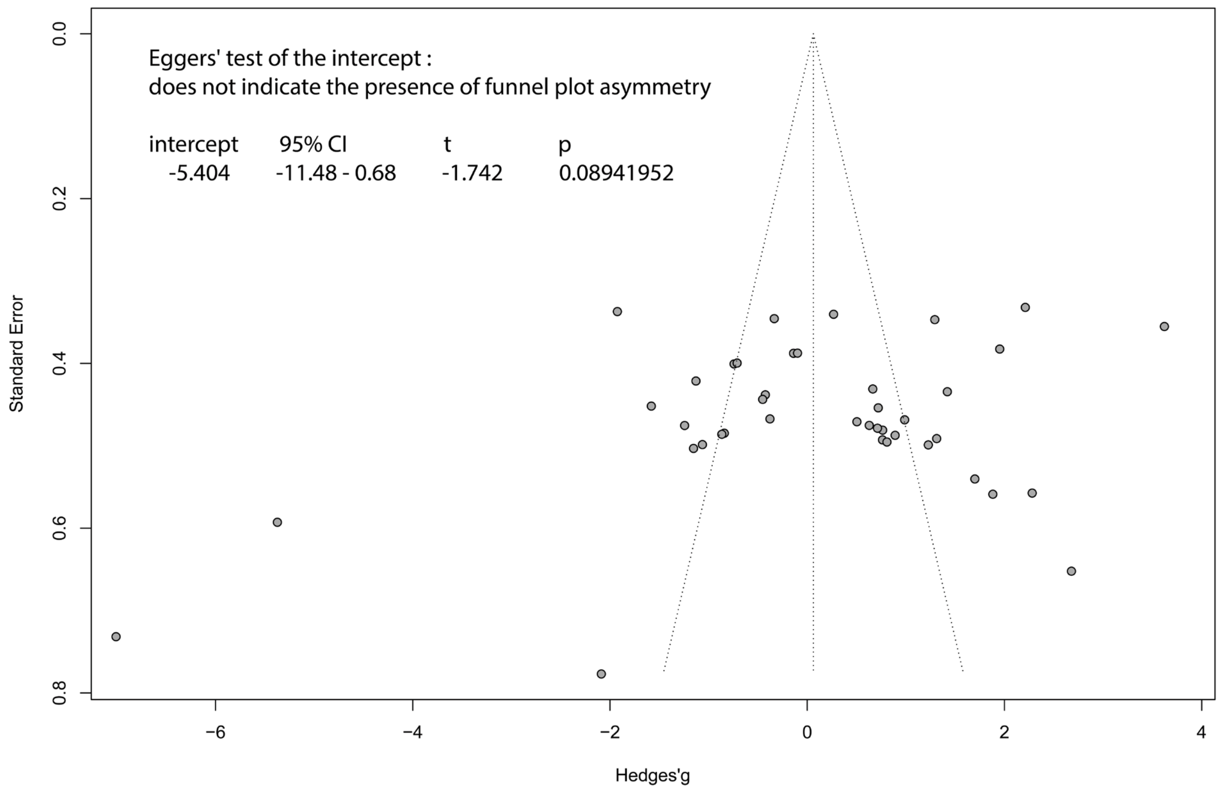

Suppl. Figure 1. Funnel plot does not indicate publication bias for studies included in the meta-analysis. Funnel plot and Eggers' test indicate the absence of publication bias.

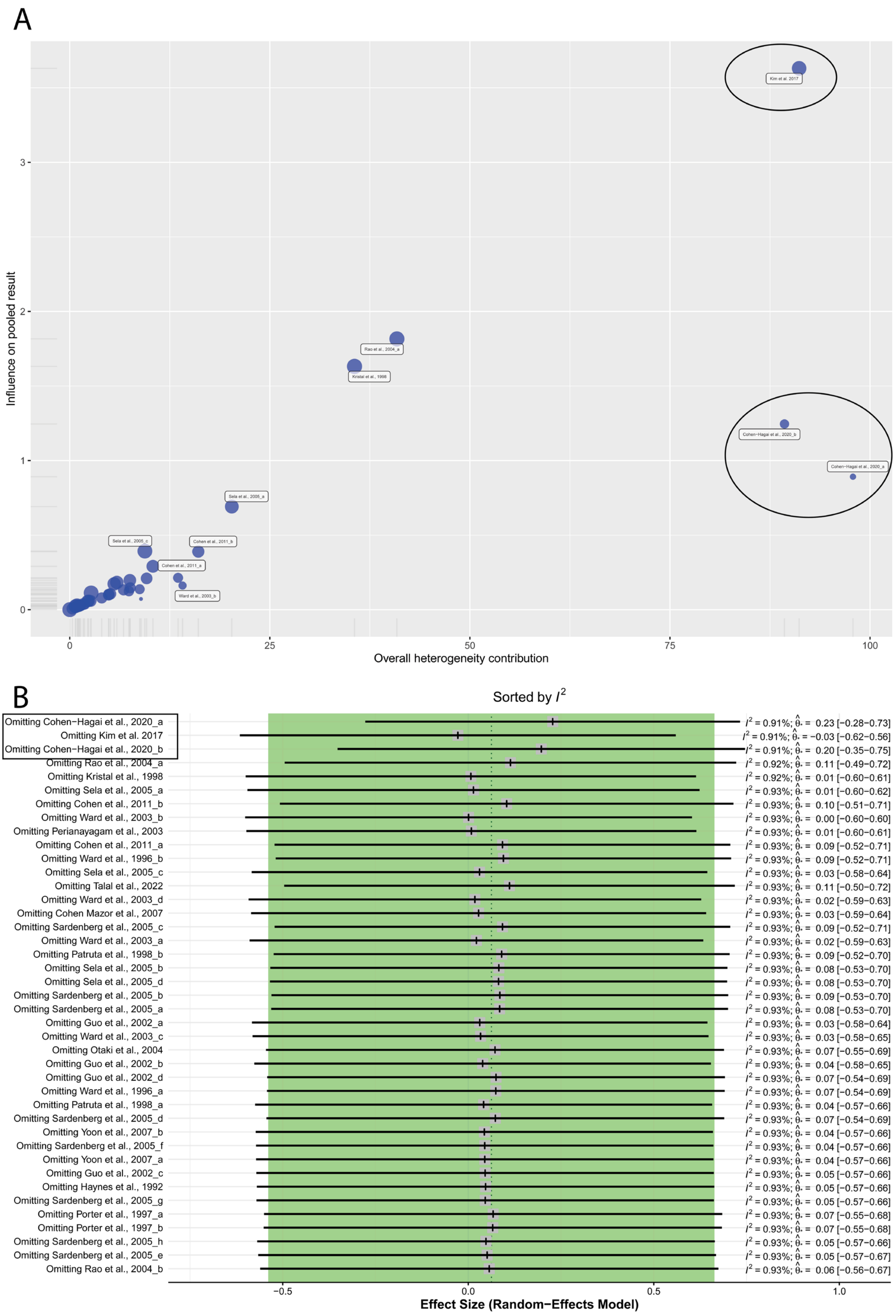

Suppl. Figure 2. Influential outlier analysis for the studies included in the meta-analysis. Influence diagnostic plots were created for the studies included in the meta-analysis, including (A) a Baujat plot; and (B) a leave-one-out analysis sorted by I^2 value. Based on a combination of these analyses, no studies were left out for the overall meta-analysis.

Author	N	CKD Mean	SD	N	Control Mean	SD	SMD	95% CI	Weight	Dialysis	Stimulus
**location = intracellular**											
Cohen et al., 2011_a	15	15.20	6.20	12	22.80	6.93	−1.13	[−1.95; −0.30]	2.5%	HD	E.coli
Cohen et al., 2011_b	15	31.40	10.84	12	64.50	28.06	−1.58	[−2.47; −0.70]	2.5%	HD	PMA
Otaki et al., 2004	17	92.90	9.48	17	95.30	2.89	−0.33	[−1.01; 0.34]	2.5%	HD	resting
Perianayagam et al., 2003	11	211.00	115.00	12	23.00	5.00	2.28	[1.19; 3.37]	2.4%	HD	resting
Porter et al., 1997_a	10	57.90	20.87	20	60.40	15.65	−0.14	[−0.90; 0.62]	2.5%	PD	S.epidermidis
Sardenberg et al., 2005_a	14	34.00	17.00	7	54.00	32.00	−0.84	[−1.79; 0.11]	2.4%	HD	resting
Sardenberg et al., 2005_b	14	164.00	130.00	7	320.00	241.00	−0.87	[−1.82; 0.09]	2.4%	HD	S.aureus
Sardenberg et al., 2005_c	14	52.00	25.00	7	103.00	66.00	−1.15	[−2.14; −0.17]	2.4%	HD	fMLP
Sardenberg et al., 2005_d	14	73.00	77.00	7	110.00	123.00	−0.38	[−1.29; 0.54]	2.4%	HD	PMA
Sardenberg et al., 2005_e	14	73.00	38.00	7	54.00	32.00	0.50	[−0.42; 1.43]	2.4%	PD	resting
Sardenberg et al., 2005_f	14	620.00	425.00	7	320.00	241.00	0.76	[−0.18; 1.71]	2.4%	PD	S.aureus
Sardenberg et al., 2005_g	14	188.00	131.00	7	103.00	66.00	0.71	[−0.23; 1.65]	2.4%	PD	fMLP
Sardenberg et al., 2005_h	14	179.00	96.00	7	110.00	123.00	0.63	[−0.30; 1.56]	2.4%	PD	PMA
Cohen−Hagai et al., 2020_a	44	18.00	1.42	15	30.00	2.33	−7.01	[−8.44; −5.57]	2.2%	HD	E.coli
Cohen−Hagai et al., 2020_b	44	64.00	5.22	15	95.00	6.95	−5.37	[−6.53; −4.21]	2.4%	HD	PMA
Ward et al., 2003_a	11	2.33	0.93	9	0.97	0.49	1.70	[0.64; 2.76]	2.4%	HD	resting
Ward et al., 2003_b	11	2.57	0.63	9	0.97	0.49	2.68	[1.40; 3.96]	2.3%	HD	resting
Ward et al., 2003_c	11	81.50	63.45	9	20.26	10.13	1.23	[0.25; 2.21]	2.4%	HD	S.aureus
Ward et al., 2003_d	11	90.62	47.33	9	20.06	10.02	1.88	[0.79; 2.98]	2.4%	HD	S.aureus
Yoon et al., 2007_a	9	18.62	7.81	9	13.23	5.43	0.76	[−0.20; 1.73]	2.4%	HD	resting
Yoon et al., 2007_b	9	17.72	7.85	9	12.90	1.70	0.81	[−0.16; 1.78]	2.4%	HD	resting
Guo et al., 2002_a	11	474.00	258.70	10	213.00	50.60	1.31	[0.35; 2.28]	2.4%	HD	resting
Guo et al., 2002_b	11	1091.00	772.77	10	468.00	332.04	0.99	[0.07; 1.91]	2.4%	HD	S.aureus
Guo et al., 2002_c	11	951.00	557.19	10	579.00	417.42	0.72	[−0.17; 1.61]	2.5%	HD	fMLP
Guo et al., 2002_d	11	1278.00	713.07	10	1635.00	806.38	−0.45	[−1.32; 0.42]	2.5%	HD	PMA
**Random effects model**	**374**			**253**			**−0.08**	**[−0.95; 0.80]**	**60.6%**		
Heterogeneity: $I^2 = 92\%$, $\tau^2 = 4.2468$, $p < 0.01$											
**location = extracellular**											
Cohen Mazor et al., 2007	20	29.73	8.54	10	18.43	5.69	1.42	[0.57; 2.27]	2.5%	HD	PMA
Haynes et al., 1992	11	15.56	9.52	12	10.06	6.24	0.66	[−0.18; 1.51]	2.5%	PD	fMLP
Patruta et al., 1998_a	10	0.69	0.32	9	0.40	0.30	0.89	[−0.06; 1.85]	2.4%	HD	resting
Patruta et al., 1998_b	10	2658.82	1361.14	9	5484.42	3409.35	−1.06	[−2.04; −0.09]	2.4%	HD	PMA
Sela et al., 2005_a	30	25.70	8.71	15	10.14	5.59	1.95	[1.20; 2.70]	2.5%	HD	PMA
Sela et al., 2005_b	20	4.19	6.57	10	15.83	25.18	−0.74	[−1.53; 0.04]	2.5%	HD	Zymosan
Sela et al., 2005_c	30	20.45	8.71	15	10.14	5.59	1.29	[0.61; 1.97]	2.5%	PD	PMA
Sela et al., 2005_d	20	5.24	3.04	10	15.83	25.18	−0.71	[−1.49; 0.07]	2.5%	PD	Zymosan
Ward et al., 1996_a	8	2.10	0.90	16	3.00	2.40	−0.42	[−1.28; 0.43]	2.5%	HD	fMLP
Ward et al., 1996_b	8	5.00	1.60	16	9.00	3.60	−1.24	[−2.17; −0.31]	2.4%	HD	fMLP + TNFalpha
Rao et al., 2004_a	205	32.30	10.02	10	52.00	13.60	−1.93	[−2.59; −1.26]	2.5%	HD	PMA
Rao et al., 2004_b	65	22.30	12.90	10	19.00	5.06	0.27	[−0.40; 0.93]	2.5%	HD	fMLP
Talal et al., 2022	6	7759.91	1706.73	6	14868.70	4104.32	−2.09	[−3.61; −0.56]	2.2%	HD	PMA
Kristal et al., 1998	30	25.78	7.50	30	10.10	6.46	2.21	[1.56; 2.86]	2.5%	HD	PMA
**Random effects model**	**473**			**178**			**0.07**	**[−0.73; 0.88]**	**34.4%**		
Heterogeneity: $I^2 = 92\%$, $\tau^2 = 1.7632$, $p < 0.01$											
**location = total**											
Kim et al. 2017	60	2811.76	312.22	30	1628.24	346.71	3.62	[2.93; 4.32]	2.5%	HD	resting
Porter et al., 1997_b	10	26.49	19.67	20	28.97	26.57	−0.10	[−0.86; 0.66]	2.5%	PD	S.epidermidis
**Random effects model**	**70**			**50**			**1.77**	**[−21.87; 25.40]**	**5.0%**		
Heterogeneity: $I^2 = 98\%$, $\tau^2 = 6.6532$, $p < 0.01$											
**Random effects model**	**917**			**481**			**0.06**	**[−0.54; 0.66]**	**100.0%**		
**Prediction interval**								**[−3.73; 3.85]**			
Heterogeneity: $I^2 = 93\%$, $\tau^2 = 3.4254$, $p < 0.01$											
Residual heterogeneity: $I^2 = 92\%$, $p < 0.01$											

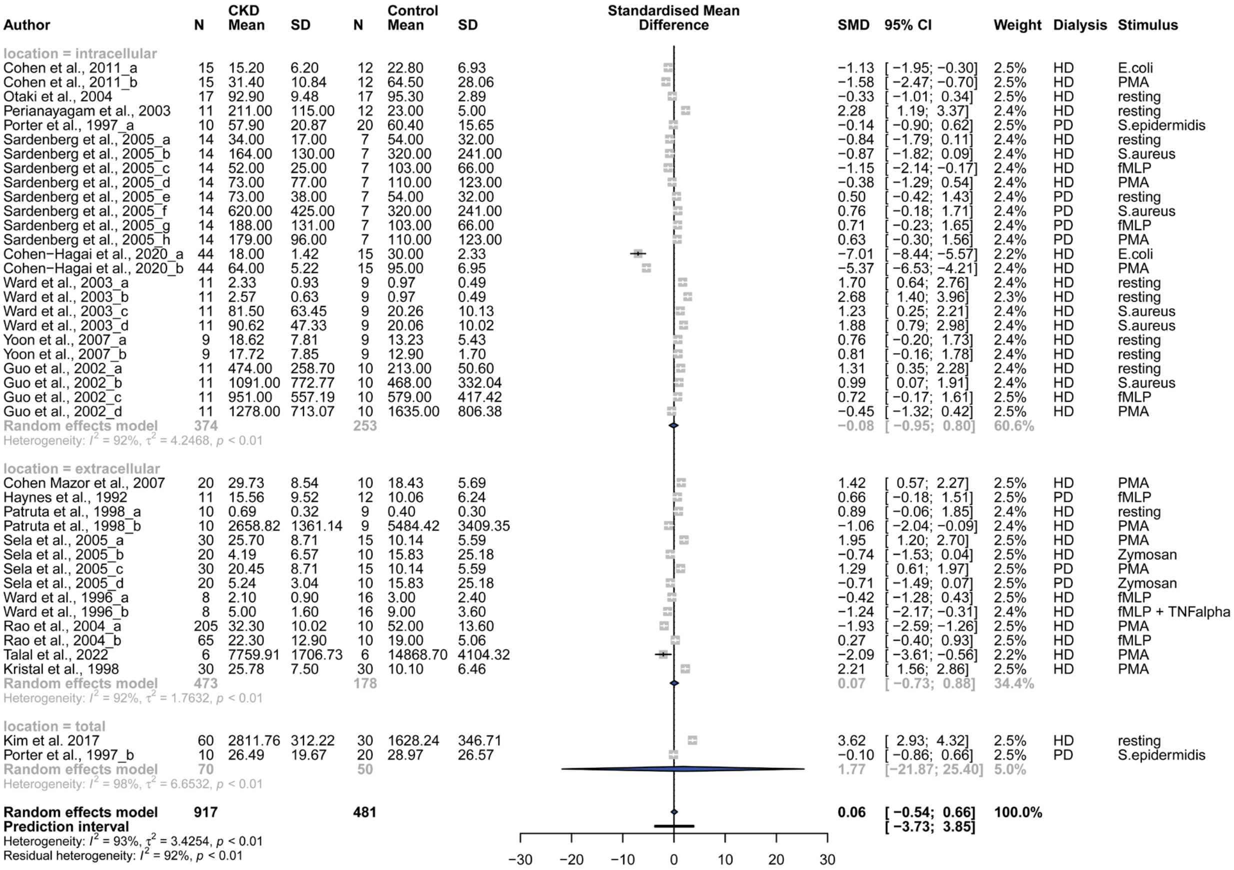

Suppl. Figure 3. Meta-analysis shows no overall significant difference in neutrophil ROS production between CKD patients on dialysis vs. controls for either intracellular or extracellular ROS. Forest plot of ROS production in neutrophils of CKD patients receiving dialysis therapy compared to those of healthy controls, with subgroup analysis for ROS location. *CKD, chronic kidney disease; CI, confidence interval; E. coli, Escherichia coli, HD, hemodialysis; fMLP, N-formyl-methionyl-leucyl-phenylalanine; PD, peritoneal dialysis; PMA, Phorbol 12-myristate 13-acetate; S. aureus, Staphylococcus aureus; SD, standard deviation, S. epidermidis, Streptococcus epidermidis; TNF*α*, tumor necrosis factor* α*.*

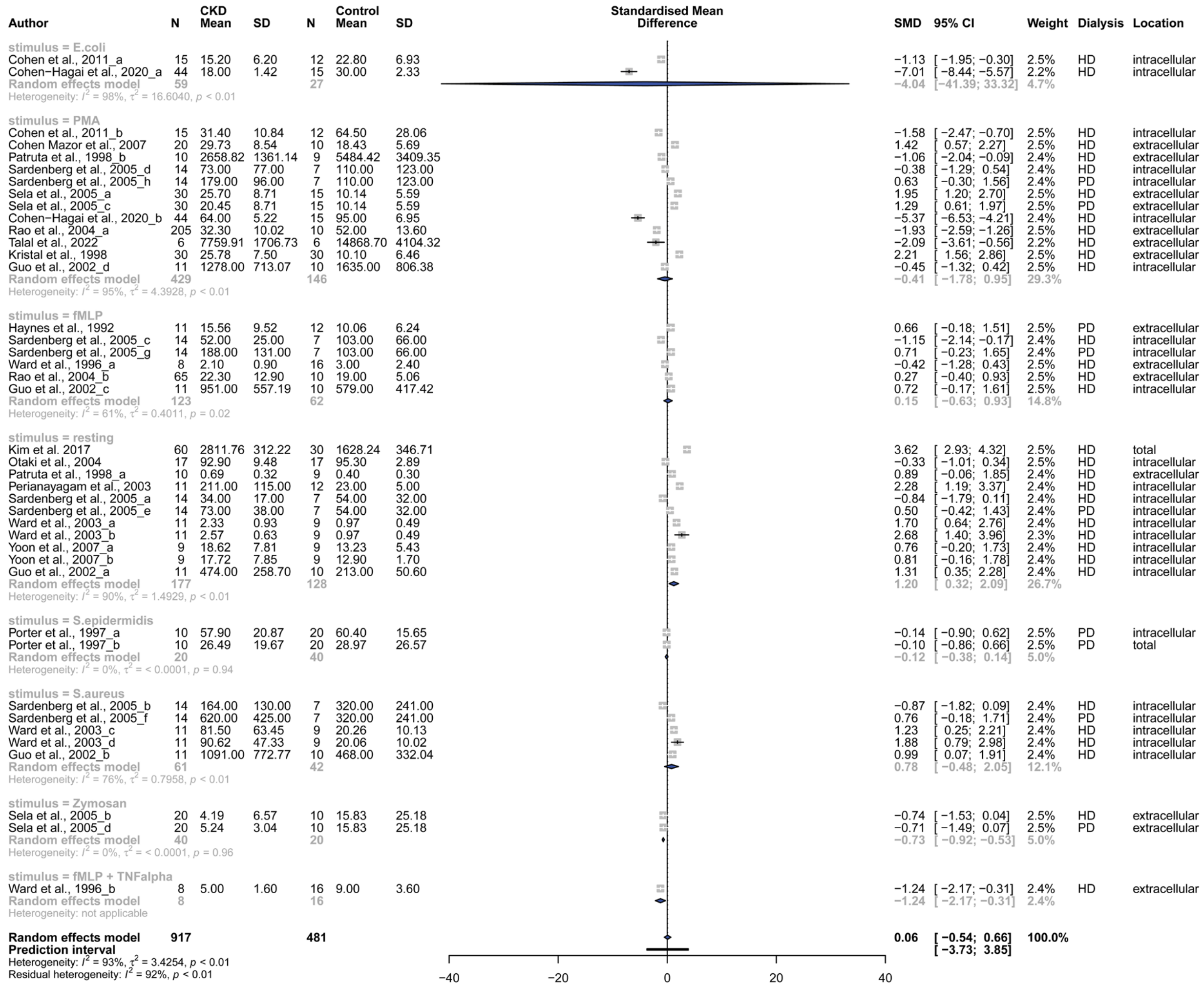

Suppl. Figure 4. Meta-analysis shows increased ROS production by resting neutrophils from dialysis patients vs. controls. Forest plot of neutrophil ROS production in dialysis patients compared to healthy controls, with subgroup analysis of effect of stimulus type on ROS generation in neutrophils. The subgroup of "resting neutrophils" is displayed in zoom in Figure 4 in the main manuscript. *E. coli, Escherichia coli, HD, hemodialysis; fMLP, N-formyl-methionyl-leucyl-phenylalanine; PD, peritoneal dialysis; PMA, Phorbol 12-myristate 13-acetate; S. aureus, Staphylococcus aureus; S. epidermidis, Streptococcus epidermidis; TNFα, tumor necrosis factor α.*
